# Lignocellulosic-Based Materials from Bean and Pistachio Pod Wastes for Dye-Contaminated Water Treatment: Optimization and Modeling of Indigo Carmine Sorption

**DOI:** 10.3390/polym14183776

**Published:** 2022-09-09

**Authors:** Gaël Ferdinand Kazé Nindjio, Rufis Fregue Tiegam Tagne, Sherman Lesly Zambou Jiokeng, Cyrille Ghislain Fotsop, Aurelien Bopda, Giscard Doungmo, Ranil Clément Tonleu Temgoua, Ingo Doench, Estella Tamungang Njoyim, Arnaud Kamdem Tamo, Anayancy Osorio-Madrazo, Ignas Kenfack Tonle

**Affiliations:** 1 Research Unit of Noxious Chemistry and Environmental Engineering, Department of Chemistry, University of Dschang, Dschang P.O. Box. 67, Cameroon; 2 Department of Paper Sciences and Bioenergy, University Institute of Wood Technology, University of Yaoundé I, Mbalmayo P.O.Box. 306, Cameroon; 3 Institut für Anorganische Chemie und Strukturchemie, Heinrich-Heine-Universität Düsseldorf, 40204 Düsseldorf, Germany; 4 Otto-von-Guericke-University Magdeburg, Chemical Institute Industrial Chemistry, Universitatsplatz 2, 39106 Magdeburg, Germany; 5 Institut für Anorganische Chemie, Christian-Albrechts-Universität zu Kiel, Max-Eyth-Str. 2, 24118 Kiel, Germany; 6 Higher Teacher Training College, University of Yaoundé 1, Yaoundé P.O. Box 47, Cameroon; 7 Laboratory for Bioinspired Materials BMBT, Institute of Microsystems Engineering IMTEK-Sensors, University of Freiburg, 79110 Freiburg, Germany; 8 Freiburg Center for Interactive Materials and Bioinspired Technologies FIT, University of Freiburg, 79110 Freiburg, Germany; 9 Freiburg Materials Research Center FMF, University of Freiburg, 79104 Freiburg, Germany; 10 Department of Chemistry, Higher Teacher Training College, University of Bamenda, Bambili P.O.Box. 39, Cameroon

**Keywords:** lignocellulosic materials, adsorption, water decontamination, Indigo Carmine, central composite design, kinetics and isotherm models

## Abstract

In this work, biomass lignocellulosic materials extracted via chemical and physical treatments from bean and pistachio pod waste were used for the optimized elimination of Indigo Carmine (IC) from aqueous medium, using a design of experiments methodology. The physicochemical properties of the studied materials (raw and treated counterparts) used for the sorption of IC were investigated by Fourier transform infrared spectroscopy (FTIR), X-ray diffraction (XRD), scanning electron microscopy (SEM) coupled with EDX, and thermal analysis. Key variables influencing the adsorption of IC, namely the initial IC concentration, the pH of the solution, the stirring time and the mass of adsorbents, were optimized by the central composite design (CCD) with three center points, the measured response being the amount of IC adsorbed. The optimal conditions obtained from the statistical analysis for the removal of IC were as follows: maximum adsorbed amounts of IC: 1.81 mg/g, 2.05 mg/g, 3.56 mg/g; 7.42 mg/g, 8.95 mg/g, 15.35 mg/g, for raw bean pods (RBS), BST1 and BST2 (bean pods chemically treated), and for raw pistachio pods (RPS), PST1 and PST2 (pistachio pods chemically treated), respectively. The pseudo-second-order nonlinear kinetics model well described the IC adsorption kinetics for RBS, BST1 and BST2, while the Elovich model was properly fitted by RPS, PST1, and PST2 biomaterials data. The Freundlich isotherm best described the shrinkage of IC on different sorbents. The good correlation of the experimental data of the IC with respect to the Freundlich isotherm indicated a multilayer adsorption with heterogeneous adsorption sites and different energies. The interest of this work consisted in developing analytical methods for the treatment of water polluted by dyes by using biosorbents, local biological materials widely available and inexpensive. The results collected in this work highlighted the interesting structural, morphological, and physico-chemical properties of the agro-waste used in the study, which properties allowed an important fixation of the target dye in solution. The research showed that the agro-waste used in the study are possible precursors to locally manufacture adsorbents at low cost, thus allowing the efficient removal of waste and dyes in liquid effluents.

## 1. Introduction

Water is perpetually polluted due to its widespread use around the world. This pollution is due to the presence in water of various pollutants, such as heavy metals, pesticides and dyes. Dyes currently occupy an important position in industrial activities and in daily life. This is the case for Indigo Carmine (IC), which is a synthetic dye of the indigoid family, presenting toxicity issues [1]. It is widely used in several fields, such as the cosmetics industry, paper mills [2], the pharmaceutical industry, the food industry [3], and the textile industry for fabric dyeing [4,5]. IC is a poorly biodegradable dye, and its uncontrolled release into the natural environment causes strong bioaccumulation. In this regard, living beings are exposed to contamination when they are in contact with wastewater contaminated by IC [6]. The maximum dose of IC in water must be less than 0.005 mg/L, according to the World Health Organization (WHO) recommendations [7]. Inhalation or ingestion of IC beyond this dose causes adverse effects on the health of living beings, namely severe hypertension, cardiovascular and respiratory effects, gastrointestinal irritation and even vomiting and diarrhea [8,9,10]. It is essential to treat wastewaters before discharge into nature. The search for solutions for the elimination of the afore-mentioned pollutants leads to the investigation of new physicochemical processes by means of adsorption, flocculation/coagulation, electrocoagulation, ion exchange principles [11,12], chemical oxidation processes (dichloride, chlorine oxide, ozone), and advanced oxidation processes (AOP) based on the production of hydroxyl radicals [13]. However, chemical oxidation has a low oxidation capacity against certain pollutants, and requires a large amount of energy. Among physicochemical processes, adsorption is the most accessible and used method, is easy to implement, and is non-polluting compared to other methods such as the coagulation/flocculation process, and electrocoagulation [14], which produce large quantities of sludge and often require prior treatment. Therefore their costs are high for industries [7]. Commercial activated carbon is the most commonly used material in the uptake of pollutants by adsorption, but, as the main drawback, it cannot be regenerated after adsorption [15,16]. To address face this drawback, there is increasing interest in adsorbents derived from the residues of legumes. Leguminous crops are essential for many reasons. They are rich in nutrients and have a high protein content. This makes them an ideal source of protein, especially in regions where meat and dairy products are not accessible for geographical or economic reasons [17,18,19]. These crops represent an important resource because they can be both sold and consumed by populations [20,21]. Pistachio pods and bean pods (*Phaseolus vulgaris L.*) are among the many other lignocellulosic materials obtained from agricultural residues and legumes [22,23,24]. These biological materials are essentially composed of macromolecules such as lignocellulose including cellulose, lignin and hemicellulose [25,26,27,28]. Due to their interesting and versatile properties, namely their abundance, renewability, easy degradation, high porosity and specific surface area, and chemical composition, rich in carbon, hydrogen, and oxygen, as well as their good mechanical properties and ease of modification, these materials represent an important pool for the production of renewable biopolymers with high absorption capacity, applicable in various fields [29,30,31,32,33,34,35,36,37,38,39]. In the last few decades, adsorbents based on the biomass of legume residues have been the subject of great attention because they are able to quantitatively adsorb recalcitrant pollutants contained in wastewater [40]. Various research works have been carried out on the sorption of dyes using agricultural biomass residues such as mango pits [41], coconut shells [42], sawdust [42], and hazelnut shells [43], etc. To date, no complete study on the recovery of this waste into biomaterials used in the context of environmental depollution has yet been carried out. On the other hand, the main organic components of bean and pistachio pods, i.e., cellulose and hemicellulose, were not used. In theory, any botanical material containing cellulose, hemicellulose, or lignin has potential use as a precursor to adsorbents [27,44,45,46].

This work aimed at the preparation and characterization of high–quality biomass absorbents from bean and pistachio pods, and their application in water bioremediation in the efficient removal of dye contaminants such as the Indigo Carmine (IC). The use of these wastes as precursors has substantial economic and environmental impacts not only by converting unwanted agricultural wastes into high-value adsorbents, but also by contributing to the economic growth of the country by reducing the import of adsorbents from international markets. Furthermore, these agricultural wastes used locally by populations for heating needs are generally incinerated. The result of the calcination of these materials is the production and release into the atmosphere of greenhouse gases that are potentially destructive to the ozone layer. One of the solutions to these various environmental problems lies in the recovery of this waste for more profitable needs. The recovery of this agricultural waste in our work offers new prospects for using these materials to solve significant environmental problems. Previous works have shown the influence of several parameters in the adsorption process, which can lead to approximate conclusions if these parameters are not effectively controlled [47]. To remove this ambiguity, response surface methodology (RSM), a powerful and mathematical statistical analysis technique, is increasingly used to assess the relative significance of several independent variables, and to determine optimal conditions for desirable responses [48]. Compared to the classical approach for the same number of estimated parameters, RSM also reduces the number of experiments to be undertaken [49,50]. To our knowledge, no work has yet been carried out on optimization by RSM for the elimination of IC using the biomass obtained from bean pods and pistachio pods. In the present study, the CCD-based RSM with three center points was adopted to optimize the removal of IC in an aqueous solution using bean pods and pistachio pods, treated on one hand with aqueous basic–acidic solvents, and on the another hand with CH_4_N_2_O+HCl+ (NH_4_)_2_S_2_O_8_. In this study, bean and pistachio pod residues were chemically treated to obtain high performance adsorbents with active adsorption sites and developed mesoporosities allowing better sequestration of dye molecules in solution. The main objective of this study was to obtain the optimal operating conditions for the elimination of IC by studying the unique and interactive effects of four significant operating variables on sorption processes: the concentration of IC in solution, the mass of biomass, the stirring time, and the pH of the IC solution. A quadratic model has been proposed to describe the relationship between removal efficiency and operating variables. Based on the nonlinear regression analysis, four kinetics models, including pseudo-first-order, pseudo-second-order, Elovich, and intraparticle diffusion, were used to infer the adsorption mechanisms. Moreover, experimental two-parameter nonlinear isothermal models were fitted with Langmuir, Freundlich, Temkin, and Dubinin-Radushkevich (D–R), and three-parameter equations were also fitted with Redlich–Peterson, Hill, Kahn, and Toth, to analyze the adsorption process.

## 2. Materials and Methods

### 2.1. Chemicals

**Scheme 1:**
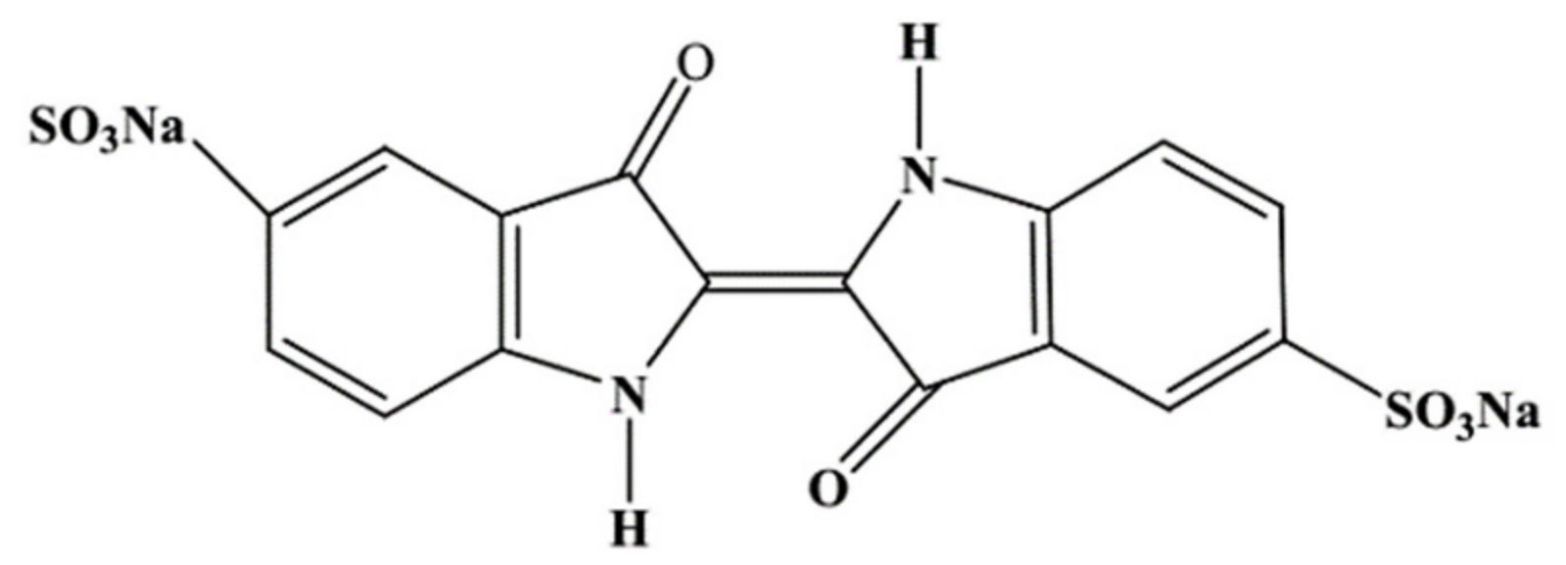
Structure of Indigo Carmine (IC).

All chemicals were used of analytical grade and without further purification. Indigo Carmine (IC, 99%) also called indigotine or acid blue 74 (Scheme 1), was purchased from Sigma-Aldrich. Test solutions were made by diluting the stock solution in distilled water. NaOH (0.10 mol/L) and HCl (0.10 mol/L) were used to adjust the pH value of the IC solution. CH_4_N_2_O, (NH_4_)_2_S_2_O_8,_ HNO_3,_ sodium thiosulfate Na_2_S_2_O_3_, and iodine solution were purchased from Sigma-Aldrich (Schnelldorf, Germany).

### 2.2. Preparation of Lignocellulose-Based Adsorbents by Different Treatments

Bean (RBS) and pistachio pods (RPS) were collected in the West Cameroon region. These raw materials were firstly washed with tap water and then rinsed with distilled water to remove impurities. The materials were cut into small pieces, again washed several times with distilled water, and finally dried. The clean and dried pods were furtherly ground and sieved to obtain particles of size less than or equal to 100 µm, before undergoing chemical treatments to extract the lignocellulosic compounds.

The lignin isolation process was carried out according to the protocol described by Brdar et al. in 2011 [51]. In the so-called basic–acidic treatment 1 of the present work, the biomass was mixed with 2 M NaOH solution and left under stirring for 24 h. After filtration, the filtrate was neutralized with a concentrated HCl solution, and the system was adjusted to pH 2. The solid precipitated after acidification was stirred for 24 h, washed with distilled water, and finally dried at 60 °C.

As for the so-called treatment 2 with urea, 10 g of biomass was mixed in 100 mL 0.1 M HCl solution with 1 mL of 1 M urea, and then stirred continuously for 24 h. Afterwards, 10 mL of a 0.1 M ammonium persulfate solution was added to the stock solution and the whole mixture was stirred for another 24 h. After filtration and washing with acetone (to remove unreacted urea oligomers and monomers), the filtrate was dried in the oven at 60 °C and stored in crucibles for later use.

The bean pod (RBS) and pistachio pod (RPS) materials obtained from the basic and acidic treatment (treatment 1) were denoted BST1 and PST1, respectively, and those resulting from the treatment with CH_4_N_2_O+HCl+(NH_4_)_2_S_2_O_8_) (treatment 2) were denoted as BST2 and PST2.

### 2.3. Adsorbents Characterization

The raw natural (bean (RBS) and pistachio pods (RPS)) and treated materials (BST1, PST1, BST2, and PST2) were characterized by various physicochemical techniques.

#### 2.3.1. Iodine Number

The American Society for Testing and Materials (ASTM D4607) standard method was used for the determination of iodine numbers according to the following protocol [52]: 0.1 g of each biomass substrate was introduced into a 100 mL Erlenmeyer flask containing a 0.02 M iodine solution and stirred for 3 h before being filtered. Subsequently, 10 mL of the obtained filtrate, containing the excess iodine, was titrated with a 0.005 N sodium thiosulfate Na_2_S_2_O_3_ solution, using starch solution as an indicator. The equivalence point was identified by the change in color from a blue–violet solution to colorless. A blank test was carried out following the same procedure.

The iodine number (mg/g) was obtained by the following formula:

(1) $$Q=\frac{C_{O}\frac{C_{T}V_{T}}{2V_{I2}}}{M_{\mathrm{ads}}}\times M_{I2}\times V$$

where V_T_ is the volume of sodium thiosulfate (mL), C_T_ the sodium thiosulfate concentration (mol/L), C_O_ the concentration of the initial iodine solution (0.02 mol/L), V_I2_ the volume of iodine dosed (10 mL), M_I2_ the iodine molar mass (253.81 g/mol), V the adsorption volume (30 mL) and M_ads_ the mass of adsorbent (g).

#### 2.3.2. pH at Zero Point of Charge pH_pzc_

The determination of the pH_pzc_ of natural materials (RBS and RPS) and treated materials (BST1, PST1, BST2, and PST2) was performed according to a method previously described in the literature [40]. First, 0.15 g of each material was added to 50 mL of 0.01 M NaCl solution, whose initial pH (pH_i_) was measured and adjusted between 1 and 12 with NaOH or HCl solutions. The containers were sealed and placed on a mechanical platform shaker for 24 h, after which the final pH (pH_f_) was measured. The pH of each tube was adjusted between 1 and 12 by adding 0.1 M NaOH or HCl solution. The pH_pzc_ corresponds to the point where the curve of ∆pH = pH_f_ − pH_i_ = f(pH_i_) crosses the line pH_i_.

### 2.4. Physicochemical Characterization

Fourier transform infrared (FTIR) spectra were registered in the 400–4000 cm^−1^ range on a Genesis spectrometer (ATI Mattson), equipped with deuterated triglycine sulfate (DTGS).

Thermogravimetric analyses were performed using a thermal analyzer (NETZSCH STA 409C/CD, Netzsch Gerätebau GmbH, Selb, Germany) at a heating rate of 10 °C.min^−1^, starting from room temperature to a maximum temperature and reaching 1400 °C under a N_2_ atmosphere.

X-ray diffraction (XRD) patterns were collected using a Stoe Stadi-P X-ray powder diffractometer (STOE & Cie GmbH, Darmstadt, Germany) operating at 40 kV and 30 mA, with the Cu Kα1 radiation (λ = 1.54056 Å, gemonochromator, flat sample). The data were collected in the 2θ angle ranging from 0° to 70°, with a scanning speed of 5° min^−1^.

The surface morphology of the materials was characterized by a scanning electron microscope (SEM) using a XL30 ESEM brand device (FEI, Hillsboro, OR, USA), with a Genesis energy dispersive X-ray detector (EDAX, Mahwah, NJ, USA). Prior to SEM imaging, the samples were sputtered with a thin gold film to improve their conductivity.

Nitrogen adsorption–desorption isotherms (BET method) were collected for selected samples using Thermo Electron Corporation, Sorptomatic Advanced Data Processing. Before N_2_ adsorption, the samples were degassed at 307.13 K under a vacuum.

### 2.5. Experimental Design for Optimization

Central composite design (CCD) was used to optimize the removal process of IC. As key parameters, the IC concentration varying between 5 and 50 mg/L, the solution pH varying between 2 and 8, the contact time varying between 5 and 60 min, and the mass of the different materials varying between 50 and 100 mg, were chosen as variables A, B, C, and D, respectively, in the CCD. The observed response Y corresponded to the quantity of IC adsorbed on the different materials. Moreover, −1 for the lower value, 0 for the value in the center, and +1 for the upper value were the variables levels coded in the experimental values. All coded and experimental variables are presented in Table 1.

In practice, the total number of tests (N) performed for a CCD consists of a test number of the factorial plane (2^k^), a test number of the star plane (2k), and a center test number (k_c_), which is used to determine experimental errors and verify the reproducibility of the results, with k being the number of variant factors. Hence, the number of tests (N) performed was determined from Equation (2) [53].

N = 2^k^ + 2k + k_c_ = 2^4^ + 2 × 4 + 3 = 27 (2)

These experiments were randomly carried out to minimize the effects of uncontrolled factors using the software Statgraphics plus version 5.0. The model obtained from the expected responses for each experiment correlated the response to the four variables during the adsorption process on the basis of a second-order polynomial equation (Equation (3)) [54,55].

(3) $$Y=\beta_{0}+\overset{n}{\underset{i=1}{\sum }}\beta_{i}x_{i}+\overset{n}{\underset{i=1}{\sum }}\beta_{\mathrm{ii}}x_{i}^{2}+\overset{n}{\underset{i=1}{\sum }}\overset{n}{\underset{j=1}{\sum }}\beta_{\mathrm{ij}}x_{i}x_{j}+\varepsilon$$

Y is the predicted response, β_0_ is the constant of the quadratic equation, β_i_ is the linear coefficient, β_ii_ is the quadratic coefficient, β_ij_ is the coefficient of the interaction between the variables x_i_ and x_j_ of the coded values of the factors, and ε is the uncertainty between measurement and predicted values. The statistical analysis of the data was carried out using the Statgraphic plus 5.0 software by statistically evaluating them according to the following criteria: (a) each factor must present a *p* value, i.e., the value of the probability lower than 5%, with a 95% confidence limit; (b) the value of the regression coefficient R^2^ should be as close as possible to 1, which means that the experimental values and the predicted values are close to each other; and (c) the proximity between the data of the predicted points and that of the experimental points must present a normal distribution to validate the hypothesis generated by the analysis of variance (ANOVA) [47,56].

### 2.6. Adsorption Experimental Studies 

Adsorption experiments were performed at room temperature and a constant stirring speed (150 rpm). For each experiment, the desired volume of IC solution of known initial concentration was mixed with the adsorbent material, at a precise pH. The dye concentration was determined by a Thermo Scientific Genesys 10S UV–Vis spectrophotometer at 610 nm wavelength. The amount (*q_e_*) of IC adsorbed per gram of material was calculated by Equation (4):

(4) $$\;q_{e}=\left(C_{i}-C_{e}\right)\frac{V}{m}$$

where *m* (mg) is the mass of the material, *C_i_* (mg L^−1^) the initial concentration of IC, *C_e_* (mg L^−1^) the equilibrium concentration, and *V* (L) the volume of the IC.

### 2.7. Equilibrium and Kinetics Modeling

To better understand the solid–liquid adsorption process of IC on different biomaterials, the experimental data were applied to four nonlinear kinetics models: the pseudo-first order, the pseudo-second order, the kinetics model of Elovich, and the intra-particle model. In addition, for the adsorption isotherm of IC, two- and three-parameters nonlinear models were studied with the experimental data. The kinetics and isothermal models studied are presented in Table 2. The higher values for R^2^ and the smaller values for x^2^ and Average Relative Error were taken into account when choosing the most suitable models. Table S1 (in the Supplementary Materials) shows the mathematical expressions of the used error functions.

### 2.8. Desorption Experimental Studies

In the desorption studies, the IC-loaded adsorbents were mixed with various desorption agents: 0.1 N HNO_3_, 0.1 N NaOH and distilled water. The desorption percentage *D* (%) of dye was calculated with Equation (5):

(5) $$D\left(\%\right)=\frac{C_{f}-C_{r}}{C_{f}}\times 100$$

where *C_f_* and *C_r_* are the initial concentration and the final concentration of IC–loaded biosorbents, respectively.

## 3. Results

### 3.1. Adsorbent Material Characterization

**Figure 1:**
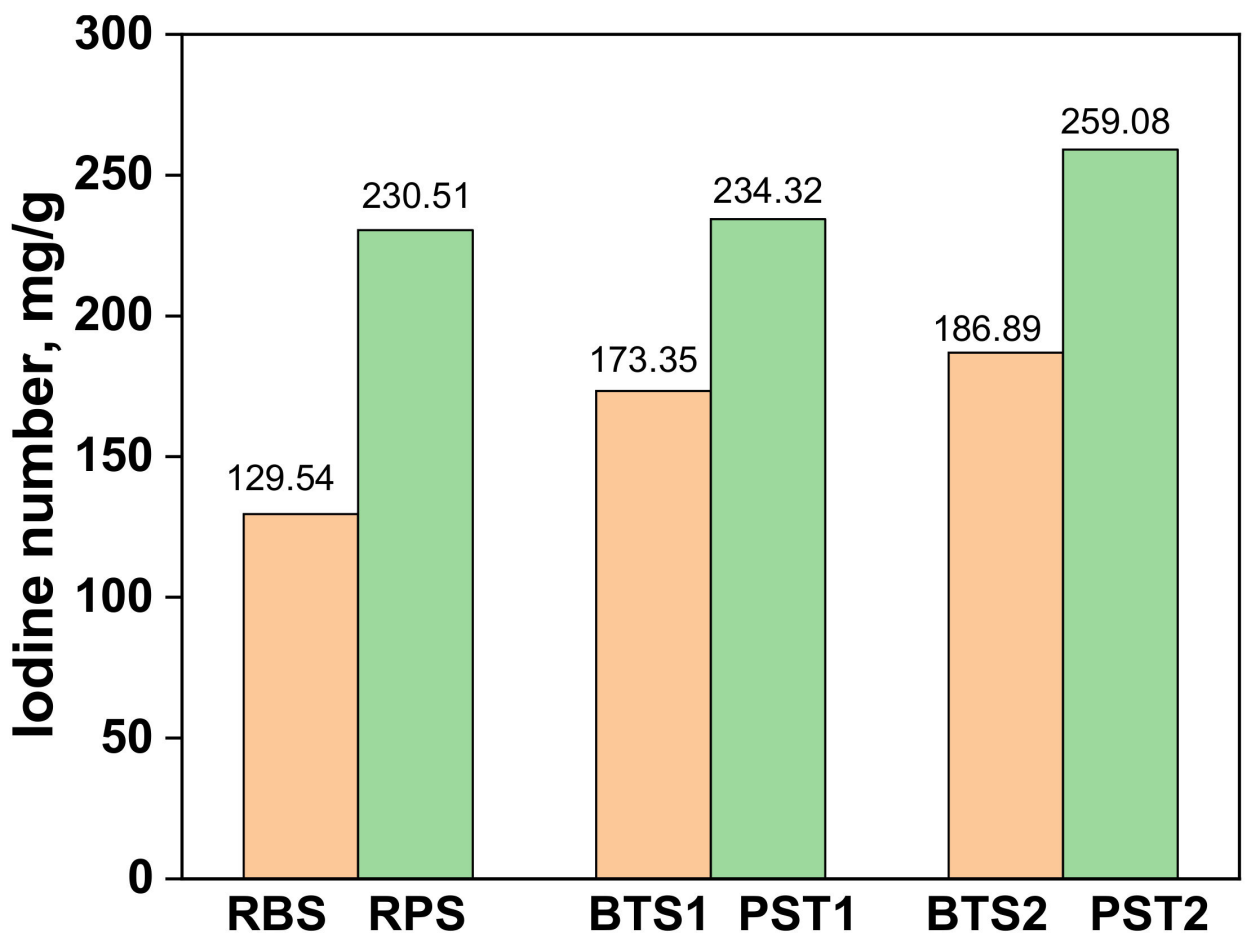
Iodine numbers recorded for raw and treated materials.

Figure 1 shows the iodine numbers of raw and treated materials. It is worth noticing the increasing variation in the iodine numbers in the raw materials with respect to the treated materials: with bean pod samples, 129.54 mg.g^−1^ for RBS to 173.35 mg.g^−1^ for BST1 and to 186 69 mg.g^−1^ for BST2. With regard to pistachio pods, values were 230.51 mg.g^−1^ for RPS, to 234.32 mg.g^−1^ for PST1 and to 259.08 mg.g^−1^ for PST2. This phenomenon should be due to the increased particle size, which leads to an increase in the number of active sites of the pores available at the surfaces of the modified materials.

The pH at zero point of charge pH_pzc_ is a good indicator of the chemical and electronic properties of functional groups. Thus, when pH_pzc_ > pH, the surface of the material is positively charged, while when pH_pzc_ < pH, the surface is negatively charged [63]. One can observe in Figure S1 (Supplementary Materials) a decrease in the pH_pzc_ from the raw material to the treated material: with bean pod samples, RBS (4.73), BST1 (3.21), and BST2 (2.38); with pistachio pod samples, RPS (4.90), PST1(4.43), and PST2 (2.40). This decrease in pH_pzc_ values can be explained by the fact that the different treatments involve the reduction of basic functions and an increase in acidic functions.

**Figure 2:**
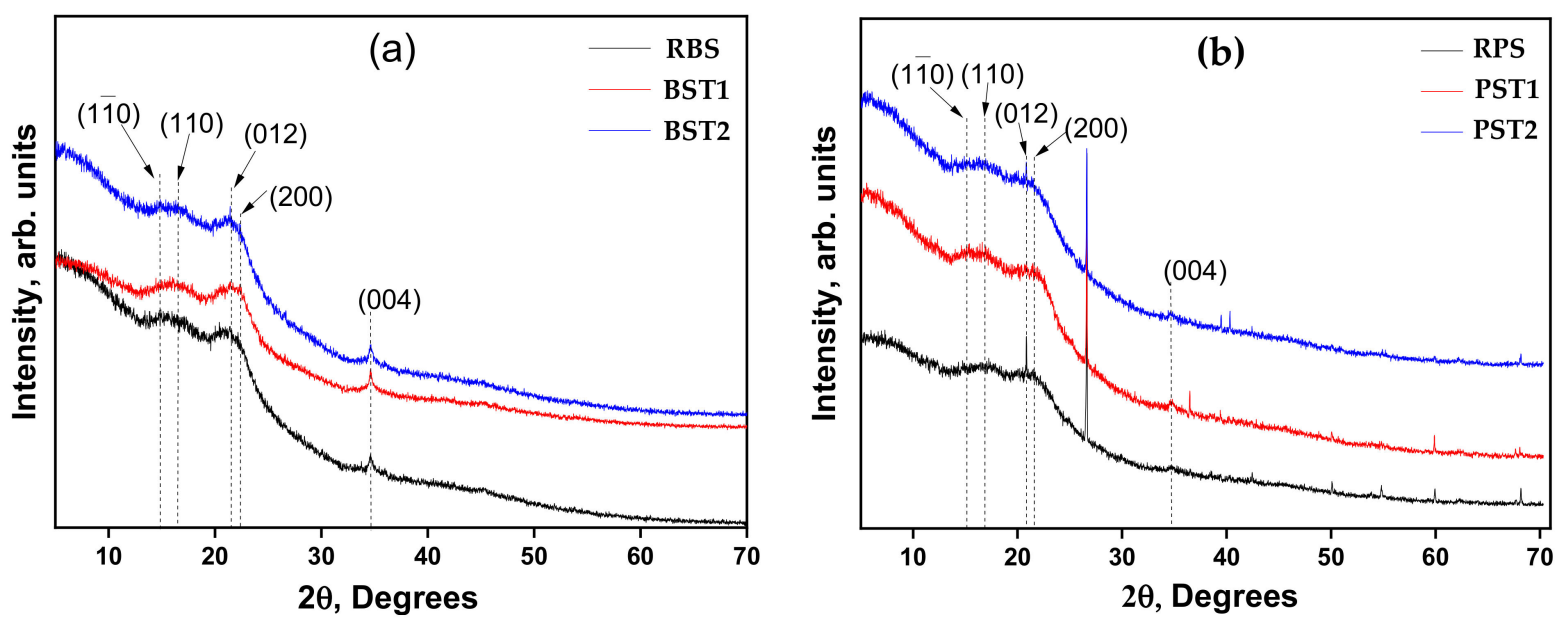
Powder X-ray diffraction (XRD) patterns of natural and treated bean (**a**) and pistachio (**b**) pod biomasses. (Black curve) Raw natural materials RBS and RPS, (red curve) BST1 and PST1 treated materials (treatment 1, basic–acidic method), (blue curve) BST2 and PST2 (treatment 2 with urea).

Figure 2 presents the XRD patterns of bean and pistachio pods samples. An amorphous structure and low crystallinity were observed for all studied materials. These patterns also displayed diffraction peaks at 2θ = 14.9°, 16.5°, 21.7°, and 34.7° corresponding to the (1–10), (110), (200), and (004) crystallographic planes of the crystal structure of cellulose I [64,65,66]. The main diffraction peaks observed in the bean and pistachio pods are summarized respectively, in Tables S2 and S3 (Supplementary Materials). Similar research showed that the diffractogram of pure cellulose highlighted crystallographic planes similar to those of the lignocellulosic biomasses studied which included mangium, pine, and candlenut shells [67]. The diffraction diagrams of different raw and treated materials show almost the same appearance and present the same diffraction peaks at 2θ = 26° which are attributed to the presence of graphite carbon in these lignocellulosic materials [68,69]. The peak at 2θ = 26°, corresponds to the highly organized layer structure of graphite, with an interlayer distance of 0.34 nm along the (002) orientation [70]. Comparison of the diffractograms of the different materials before and after modification indicates that the crystal/amorphous structure remained stable after treatment.

**Figure 3:**
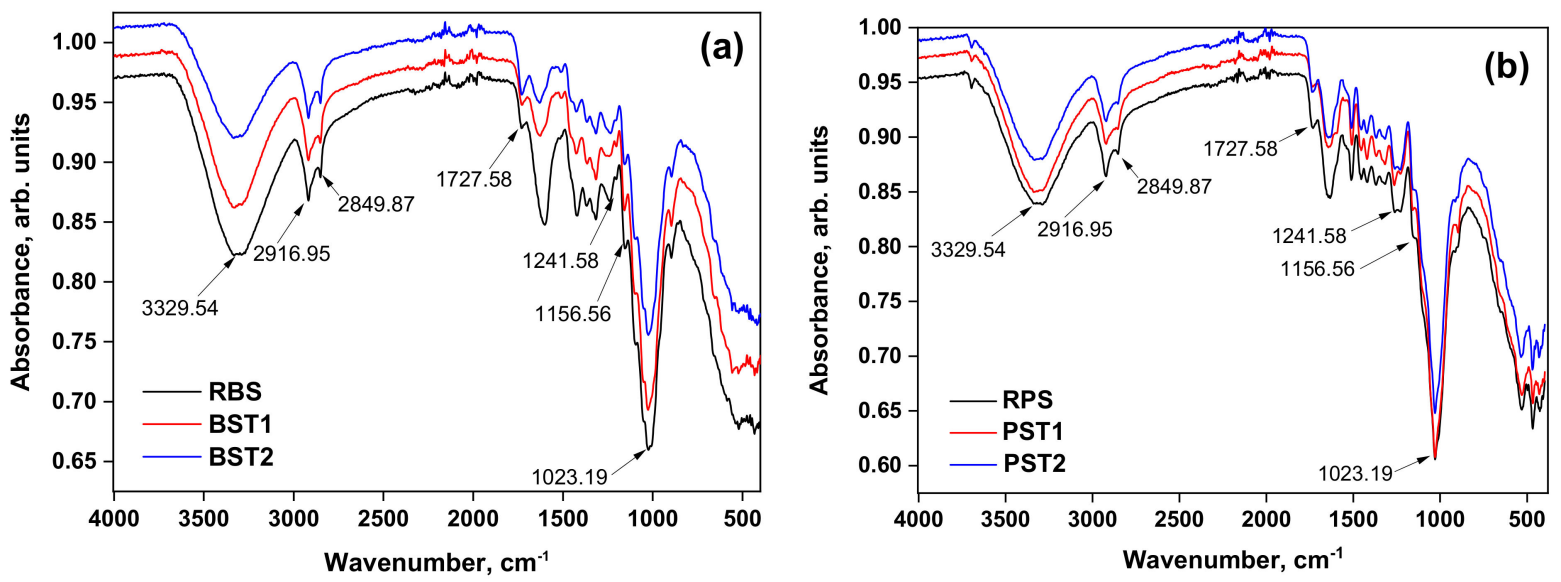
FTIR spectra of bean pods (**a**) and pistachio pods (**b**): RBS and RPS (black curve), BST1 and PST1 (red curve) and BST2 and PST2 (blue curve).

Although X-ray diffractograms did not show significant differences, FTIR spectroscopy characterizations were performed to check the chemical functions present within the structures of the six biomass samples. The FTIR spectra, shown in in Figure 3, revealed the presence of several absorption bands of valence vibrations and deformations attributable to different functional groups existing in these materials. The main vibration bands were attributed based on previously published works [71]. The spectra of raw and treated bean pods (Figure 3a) as well as those of raw and treated pistachio pods (Figure 3b), had the same shape, proof that these materials had identical chemical groups. On the spectra of the different materials, there was a wide band in the 3500–3000 cm^−1^ region centered around 3329.54 cm^−1^, characteristic of the stretching vibration of the OH groups of phenols, carboxylic acids or alcohols, and water present in hemicellulose, cellulose and lignin [8,72,73,74]. In the 3000–2500 cm^−1^ region, a double peak in the different materials was observed, centered around the point 2916.95 cm^−1^ on the one hand and 2849.87 cm^−1^ on the other hand corresponding to the elongation band characteristic of low–intensity symmetric or asymmetric C–H stretching vibration of aliphatic methyl -CH_3_ and methylene -CH_2_- groups, as observed in hemicellulose, cellulose and lignin [75]. In the region 2000–1500 cm^−1^, peaks were observed at 1727.50 cm^−1^ for the various materials, which are characteristic of the carbonyl group (C=O) of carboxylic acids [66,76]. We also noted, on the spectra, peaks centered around 1509.11 cm^−1^ for the various treated materials, which were attributed to the asymmetric elongation vibrations of the C=C double bond of the aromatic skeleton of lignin [77]. The adsorption peaks centered at 1241.58 cm^−1^ for the various raw and treated materials may be due to the stretching vibrations of the C–O, C–H or C–C bonds of the carboxylic groups (-COOH). Finally, bands in the 1200 and 1000 cm^−1^ regions and centered around 1156.56 cm^−1^ and 1023.19 cm^−1^ were assigned to the vibrations of the C–O bonds of carboxylic acids, phenols, ethers, alcohols, and aliphatic esters or amines, characteristic of the cellulosic structure of the raw material [68,78,79].

**Figure 4:**
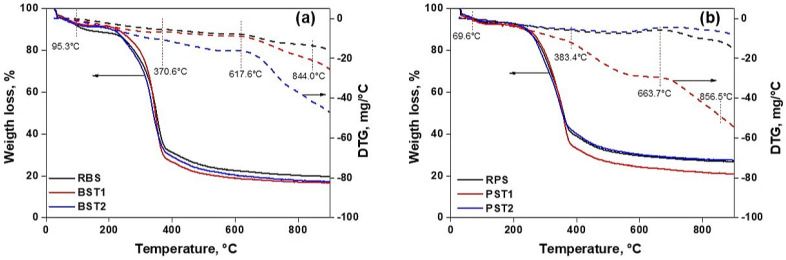
TG-DTG curves of bean pods (**a**) and pistachio pods (**b**): RBS and RPS (black curve), BST1 and PST1 (red curve), and BST2 and PST2 (blue curve). The dotted curves are those of the differential thermogravimetric analysis (DTG) for each studied sample. These thermal analyses, in which the rate of change in weight of the material upon heating is plotted against temperature, are derivatives of TGA thermogravimetric analyses.

The thermal analysis characterization of RBS, RPS, BST1, PST1, BST2, and PST2 materials was also performed and the results are shown in Figure 4. The TGA-DTA curves of the raw RBS materials, BST1 and BST2 (Figure 4a) allow us to observe, at first sight, a very slight loss of mass of 1.98% over a temperature interval of 33.8 °C–95.3 °C, corresponding to the loss of water molecules contained in the material. A mass loss of 6.25%, 7.56%, and 11.86%, respectively, for the RBS, BST1, and BST2 materials over a temperature interval of 95.3 °C–370.6 °C, was observed, corresponding to the elimination of hemicelluloses for the three materials. The high mass losses of the treated materials BST1 (7.56%) and BST2 (11.86%), compared to that of the raw material RBS (6.25%), makes it possible to affirm that the treatment with NaOH+HCl for the BST1 material and CH_4_N_2_O+HCl+(NH_4_)_2_S_2_O_8_ for the BST2 material led to an increase in the specific surface area and in the hydrophilic functional groups present on the surfaces of the materials [80]. Then for temperatures between 370.6 °C and 617.6 °C, the loss of mass of the residues of the organic fragment due to the thermal alteration of the celluloses at 10.84%, 12.34%, and 20.28%, respectively, for raw RBS, BST1, and BST2 materials, was observed. Finally, the significant mass losses at 15.56%, 23.75%, and 47.08% respectively, for raw RBS, BST1, and BST2 materials over an interval of 617.6 °C-844.0 °C were observed. This temperature interval would correspond to the breaking of the hydroxyl and amine groups present in the different materials treated [81]. Haldar & Purkait have shown that chemical treatments applied to lignocellulosic biomass can influence the thermal properties of the resulting materials. They highlighted the differences in the thermal behavior of the biomass components in relation to the severity of the alkaline pretreatment that was applied to the biomass [82]. Regarding pistachio pods (Figure 4b), the increase in the first mass loss of PST1 (11.70%) compared to that of PST2 (5.74%) may be due to the hydrophilic nature of the material after the fixation of the ammonium persulfate and urea molecules. The mass losses observed in the temperature ranges 383.4–663.7 °C and 663.7–856.5 °C corresponded, respectively, to the loss of organic fragments and the elimination of phenol functions [7]. These studies also confirm the presence of the various functional groups observed in FTIR.

**Figure 5:**
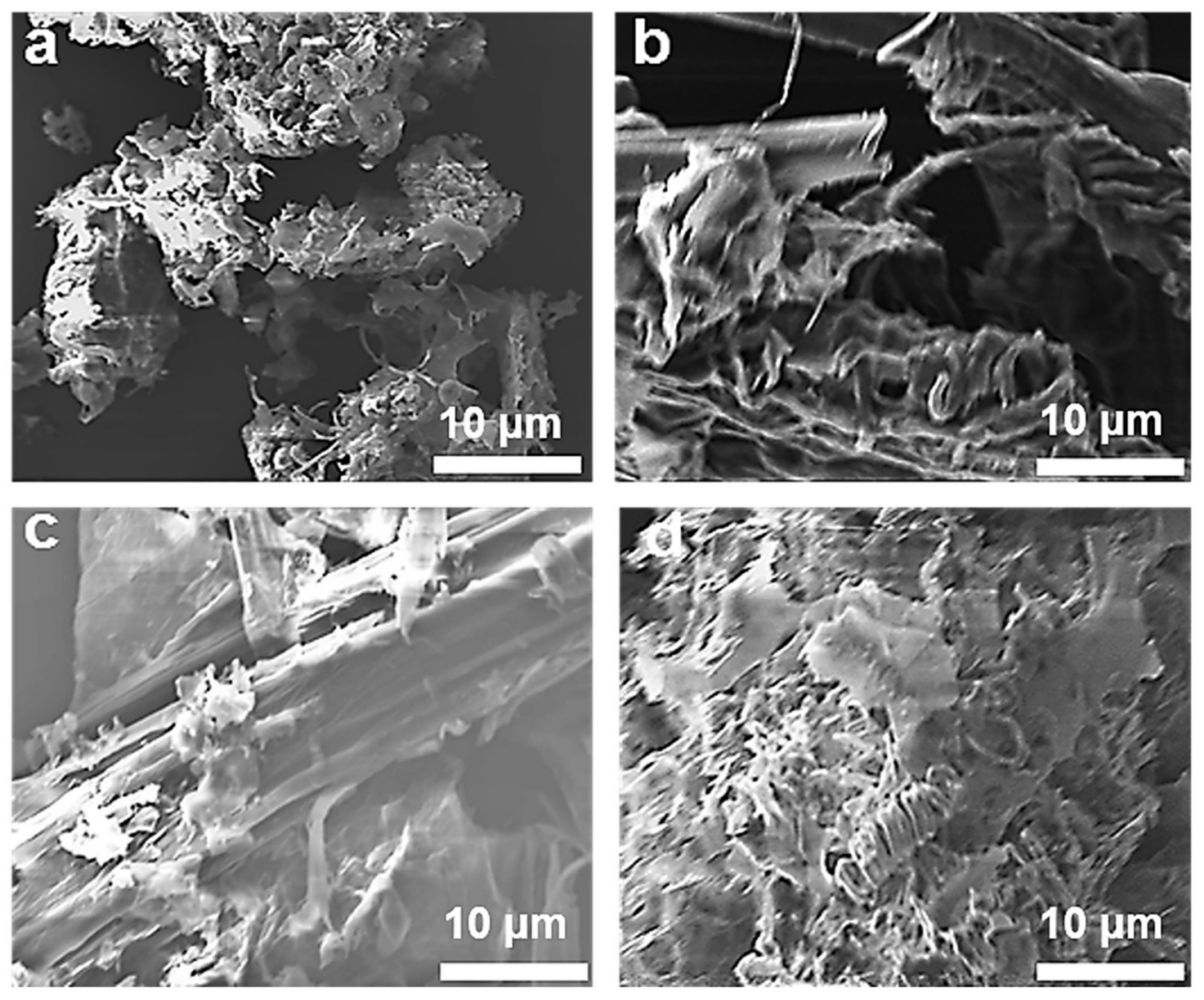
SEM micrographs of biomass materials: (**a**) RBS, (**b**) BST2, (**c**) RPS, and (**d**) PST2. Scale bar: 10 µm.

The surface morphologies of the materials RBS, RPS, BST2, and PST2 were investigated by scanning electron microscopy (SEM). The high–magnification micrographs in Figure 5 reveal a porous microstructure for all prepared biomass materials, with continuity of the cavities within the pores at different length scales. A developed porosity makes possible an increase in the specific surface of the material and, consequently, the number of active sites on which the target molecules can possibly bind. The results show that the treatment operation induces the consequent development of the porosity via the bursting of the pores naturally existing in the biomass or induced by the treatment. The SEM image shows relatively more homogeneous, wide, slit-shaped pores with a small number of large–diameter pores (mesopores). The morphology of the materials shows a different type of pores which are characterized as capillary cracks and some grains of different sizes in large holes, which are present on the surfaces of materials, the latter have homogeneous pores in the form of a wide slit. Furthermore, EDS was carried out showing the elemental compositions of the bean and pistachio pods samples (raw and treated) (Figure S2, Supplementary Materials), which verified the presence of C and O. Additionally, the atomic percentages were described in the table of elemental compositions. The high carbon content allows us to suggest that these materials could be good candidates as support materials in pollutant adsorption processes.

The specific surface area, pore volume, pore area, and pore radius of RBS, RPS, BST1, PST1, BST2, and PST2 materials were calculated from the nitrogen adsorption–desorption isotherms, via the Brunauer–Emmett–Teller (BET) and the Gurvich methods. The results obtained are shown in Table 3. The specific surface area, pore volume, pore area and pore radius of the biosorbents increased when moving from raw materials (RBS and RPS) to materials treated with NaOH + HCl (BST1 and PST1) and then with CH_4_N_2_O + HCl + (NH_4_)_2_S_2_O_8_ (BST2 and PST2). The increase in pore radius, adsorbed nitrogen volume, specific surface area, pore volume, and pore area of RBS and RPS after NaOH + HCl and CH_4_N2O + HCl + (NH_4_)_2_S_2_O_8_ treatments can be explained by the fact that the treatments with NaOH + HCl and CH_4_N_2_O + HCl + (NH_4_)_2_S_2_O_8_ lead to the withdrawal in the material of sugars, extractables, and lignin which usually block the binding sites of the material, thus favoring the development of porosity [83]. In view of these results, it was concluded that the treatment had an indispensable effect insofar as it improved the surface properties of the materials for possible applications in the process of eliminating organic and inorganic pollutants.

### 3.2. Optimization of Adsorption Parameters

In order to study the effects of four independent variables on the removal efficiency of IC, 27 designed batch runs were performed according to CCD and the results along with the experimental conditions are presented in Table S4 (for RBS/IC, BST1/IC and BST2/IC) and Table S5 (for RPS/IC, PST1/IC, and PST2/IC). A clear observation from these tables is that the experimental values (Obs value) are closed to those fitted (Fit value) by the software. From the ANOVA, an approximate function of removal efficiency based on the experimental results was evaluated and given in the empirical mathematical model that was used to examine the correlation between predicted values and experimental values. These quadratic model equations represent the fit between coded variables including initial concentration (A), solution pH (B), contact time (C), biomaterial mass (D), and predicted response (Y). We denoted Y_RBS_, Y_BST1_, Y_BST2_, Y_RPS_, Y_PST1_ and Y_PST2_ as the responses of the adsorbed quantities of IC, respectively, by RBS, BST1, BST2, RPS, PST1, and PST2.

(6) $$Y_{\mathrm{RBS}}=1.649+0.065A-0.484B-0.003C-0.023D-0.00008A^{2}-0.004\mathrm{AB}-0.0001\mathrm{AC}-0.0003\mathrm{AD}+0.033B^{2}+0.0007\mathrm{BC}+0.002\mathrm{BD}+0.00004C^{2}-0.000008\mathrm{CD}+0.0001D^{2}$$

(7) $$Y_{\mathrm{BST}1}=1.221+0.053A-0.655B+0.005C+0.0003D-0.000003A^{2}-0.005\mathrm{AB}+0.00002\mathrm{AC}-0.0002\mathrm{AD}+0.055B^{2}-0.00008\mathrm{BC}+0.0014\mathrm{BD}-0.0001C^{2}+0.00001\mathrm{CD}-0.00005D^{2}$$

(8) $$Y_{\mathrm{BST}2}=2.416+0.063A-0.964B+0.013C-0.011D+0.00006A^{2}-0.005\mathrm{AB}+0.0001\;\mathrm{AC}-0.0003\;\mathrm{AD}+0.073{\;B}^{2}-0.0009\mathrm{BC}+0.002\mathrm{BD}-0.00004C^{2}-0.00008\mathrm{CD}+5.92593\times 10^{-7}D^{2}\;$$

(9) $$Y_{\mathrm{RPS}}=10.527+0.182A-2.214B+0.088C-0.208D-0.0008A^{2}-0.010\mathrm{AB}+0.0009\mathrm{AC}-0.0004\mathrm{AD}+0.195B^{2}-0.0003\mathrm{BC}+0.0035\mathrm{BD}-0.002C^{2}+0.00034\mathrm{CD}+0.0012D^{2}\;$$

(10) $$Y_{\mathrm{PST}1}=8.561+0.195A-2.303B+0.011C-0.072D-0.001A^{2}-0.017\mathrm{AB}+0.0002\mathrm{AC}+0.0003\mathrm{AD}+0.153B^{2}-0.0002\mathrm{BC}+0.008\mathrm{BD}-0.00002C^{2}-0.00004\;\mathrm{CD}+0.00008D^{2}\;$$

(11) $$Y_{\mathrm{PST}2}=8.960+0.306A-3.856B+0.098C-0.073D-0.0006A^{2}-0.029\mathrm{AB}+0.0006\mathrm{AC}-0.0008\mathrm{AD}+0.311B^{2}-0.006\mathrm{BC}+0.012\mathrm{BD}-0.0004C^{2}-0.0006\mathrm{CD}+0.0002D^{2}\;$$

The expressions of these equations have coefficients of opposite signs. The positive sign implies a synergistic effect on the response. Therefore, a contribution to the increase in the adsorbed quantity of IC implies an increase in the factor concerned. In addition, a negative sign coefficient leads to an antagonistic effect on the response, i.e., a reduction in the adsorbed quantity of IC leads to an increase in the factor concerned. The analysis of variance (ANOVA) enabled us to identify the relationships between the quantities adsorbed on the different variables (Tables S6 to S8). A factor is said to be significant when, at the 95% confidence interval, the probable value (*p*-value) is less than 5% and R^2^ is substantially equal to one. Regarding the adsorption of IC by the different biosorbents (RBS, BST1, BST2, RPS, PST1, and PST2), we noted the presence of individual variables as well as multiple interactions that had influences on the quantity adsorbed. As for the RPS material, the individual variables A, B, and D, as well as the interactions AB, AD, BB, and BD have a significant influence on the amount adsorbed. For the BST1 material, the factors A, B, and D and the interactions AB and BB significantly influenced the quantity adsorbed. For the BST2 material, the factors A, B, and D and the interactions AB, AD, BB, and BD significantly influenced the quantity adsorbed. For the RPS material, the factors A, B, C, and D and the interactions AB, AC, AD, BB, and CC significantly influenced the amount adsorbed. Then, for the PST1 material, the factors A and B and the interactions AB, BB, and BD significantly influenced the quantity adsorbed. Finally, for the PST2 material, the factors A and B and the interactions AB, BB and BD significantly influenced the quantity adsorbed. Statistically, by removing the insignificant terms, the quadratic models lead to Equations (12)–(17).

(12) $${Y^{\prime }}_{RBS}=1.649+0.065A-0.484B-0.023D-0.004\;AB-0.0001\;AC-0.0003AD+0.033B^{2}+0.002BD\;$$

(13) $${Y^{\prime }}_{BST1}=1.221+0.053A-0.655B+0.0003D-0.005AB+0.055B^{2}$$

(14) $${Y^{\prime }}_{BST2}=2.416+0.063A-0.964B-0.011D-0.005AB-0.0003\;AD+0.073\;B^{2}+0.002BD\;$$

(15) $${Y^{\prime }}_{RPS}=10.527+0.182A-2.214B-0.010AB+0.195B^{2}+0.0035BD\;$$

(16) $${Y^{\prime }}_{PST1}=8.561+0.195A-2.303B-0.017AB+0.153B^{2}+0.008BD\;$$

(17) $${Y^{\prime }}_{PST2}=8.960+0.306A-3.856B+0.098C-0.073D-0.029AB+0.0006AC-0.0008AD+0.311B^{2}-0.006BC-0.0004C^{2}\;\;$$

These so-called reduced Equations (12)–(17) can be translated into response surfaces in order to identify areas of interest and therefore to determine optimal conditions. The experimental data were statistically analyzed by analysis of variance and the results are presented in the Tables S9–S11.

**Figure 6:**
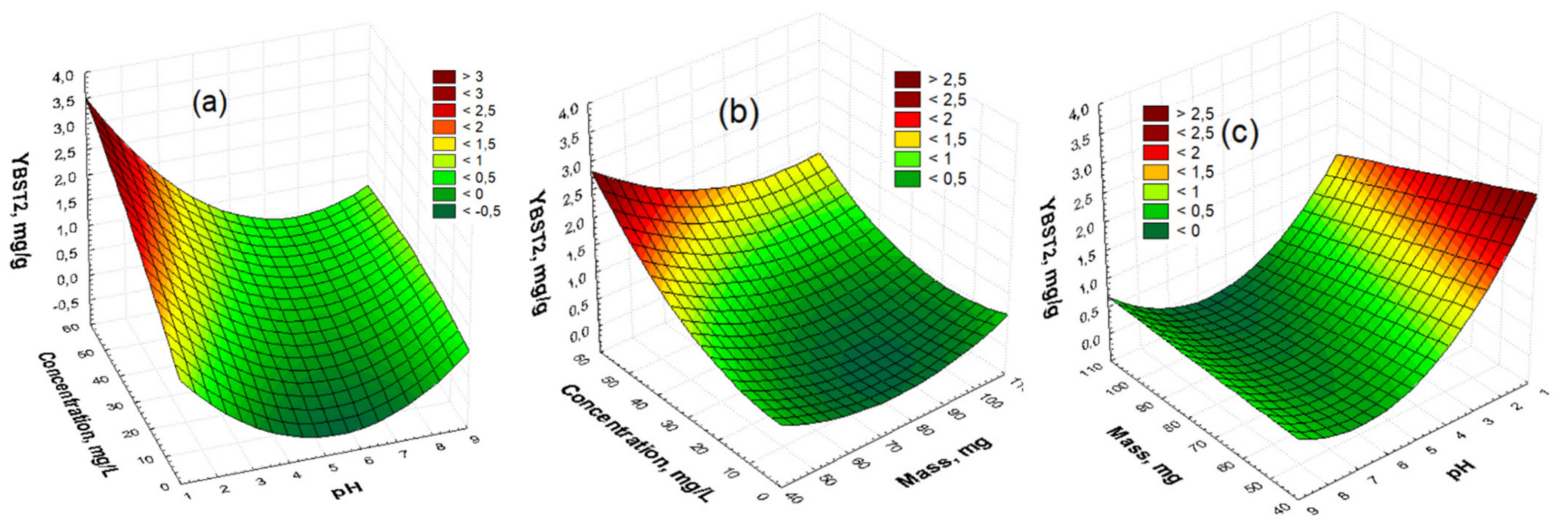
Surface response for the amount of IC adsorbed by the urea-treated (treatment 2) bean pod biomass BST2 as a function of concentration and pH effects (**a**), concentration and mass effects (**b**), pH and mass effects (**c**).

**Figure 7:**
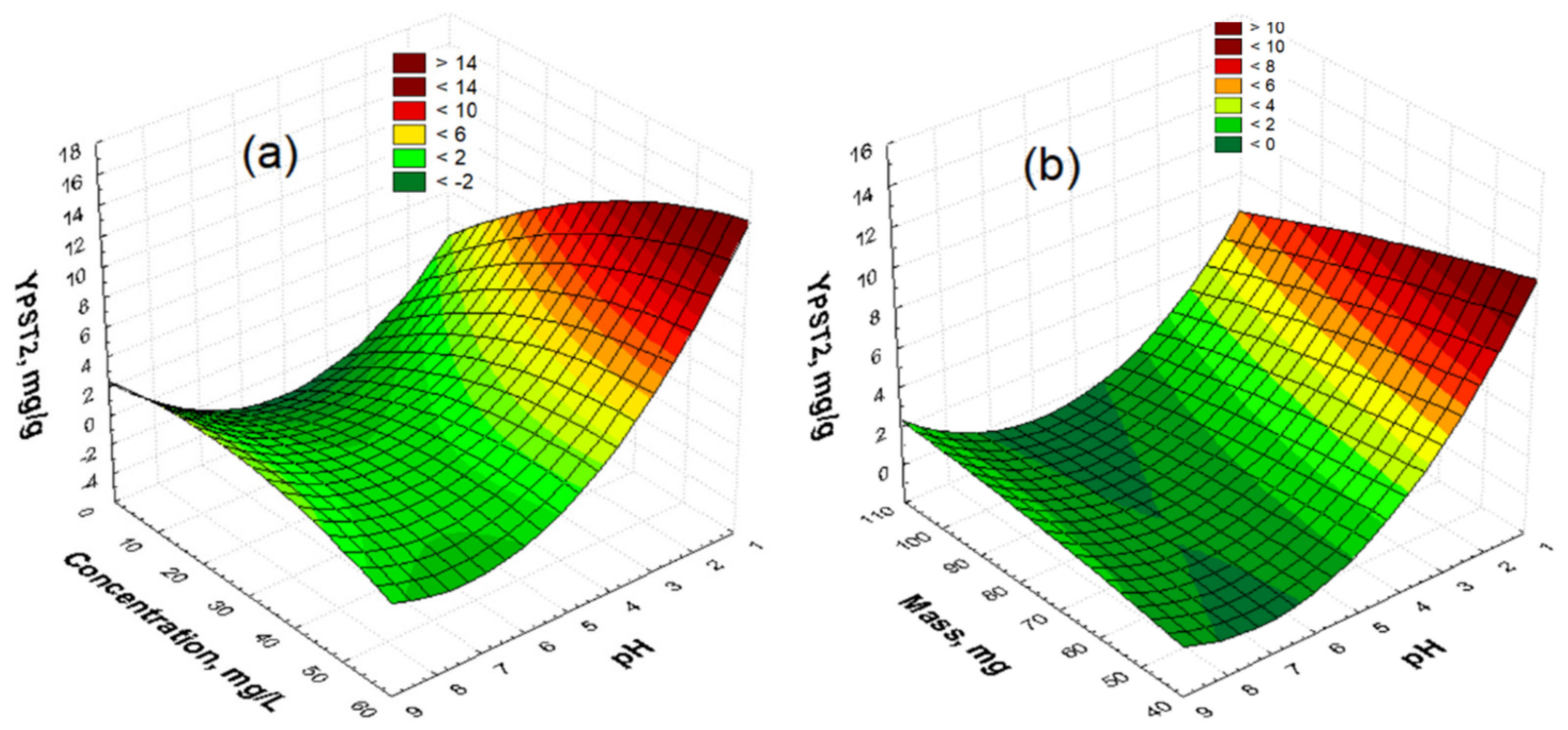
Surface response for the amount of IC adsorbed by the urea-treated (treatment 2) pistachio pod biomass PST2 as a function of concentration and pH effects (**a**), pH and mass effects (**b**).

To further assess the applicability of the quadratic model, we studied the effects of the interactions between the various independent factors A, B, C, and D according to the response measured Y by the diagrams of Pareto (Figures S3–S8) and surfaces. The results are presented in Figure 6 and Figure 7 and Figures S9–S12 for the two materials, respectively, according to their processing modes. These three-dimensional surface plots represent the impact of the optimization of the initial concentration, solution pH, contact time, and material mass on response, which is the adsorbed amount of IC. These plots depict the most important interaction factors. As regards the RBS material (Table S6), it was observed that the concentration–pH, concentration–mass, and pH-mass, interactions with a probability *p* = 0.0001; *p* = 0.0041 and *p* = 0.0134, were, respectively, significant for IC adsorption. These interactions are represented by Figure S9. For the BST1 material (Table S7 and Figure S10), the concentration–pH interaction (with *p* = 0.0000) was significant for the adsorption of IC. For the BST2 material (Table S8, Figure 6), it was observed that the concentration–pH, concentration–mass and pH–mass interactions with *p* = 0.0008, *p* = 0.0217, and *p* = 0.0151, respectively, were significant for IC adsorption. For the RPS material (Table S9, Figure S11), it was observed that the concentration–pH and concentration–time interactions, with *p* = 0.0063 and *p* = 0.0219 respectively, were significant for IC adsorption. For the PST1 material (Table S10, Figure S12), it was noticed that the concentration–pH and pH–mass interactions with a probability *p* = 0.0000 and *p* = 0.0045, were respectively significant for the adsorption of IC. For the PST2 material as shown in Figure 7, it was observed that the concentration–pH and pH–mass interactions, with a probability *p* = 0.0001 and *p* = 0.0224, were respectively significant for the adsorption of IC.

With regard to the different Pareto diagrams (Figures S3 to S8), we can physically observe that the different interactions have standardized effects, which have a positive and major impact on the quantity of IC adsorbed. Note that the adsorbed amount of IC onto RBS increases with increasing initial concentration (Figure S9a,b) and increases with decreasing pH (Figure S9c). On the BST1 material (Figure S10a), we note that the adsorbed amount of IC increases with increasing initial concentration. The adsorbed amount of IC onto BST2 increases with decreasing pH (Figure 6a,c) and the adsorbed amount of IC increases with increasing initial concentration (Figure 6b). The adsorbed amount of IC onto RPS increases with increasing initial concentration (Figure S11a,b). The adsorbed amount of IC increases for the PST1 material with decreasing pH (Figure S12a,b) and finally, the adsorbed amount of IC onto the PST2 material increases with decreasing pH (Figure 7a,b). The increase in the amount of adsorption with increasing initial concentration is due to the increase in IC molecules in the solution creating competition for different adsorption sites. According to the CCD, the maximum adsorption is at pH 2, according to the statistical analysis. At this pH, the surfaces of the various materials are charged with protons. IC is stable in a strongly acid medium and its adsorption is maximal in this medium. In all figures, we observe a decrease in the amount of IC adsorbed with the increase in mass from 50 to 100 mg. This is explained by the formation of clusters of particles of RBS, RBS, BST2, BST2, RPS, PST1, and PST2, respectively, which induces a reduction in the total surface adsorption and therefore a decrease in the amount of adsorbate per unit mass [53]. The optimal conditions for IC on the different biomasses were deduced from the maximum quantities retained. For bean waste biomasses, the best adsorption results were obtained for the urea-treated biomass (BST2) (Figure 6), but these results were lower than those obtained with all pistachio waste biomasses. Specifically, for pistachio waste biomasses, the best results were also obtained with the urea-treated pistachio pods (PST2) (Figure 7).

The experimental values of the responses were analyzed by the Statgraphics plus 5.0 software and the results are presented in Table 4. The values of the operating parameters led to the optimal results. An experiment on the adsorption of the IC by the different biomasses (RBS, BST1, BST2, RPS, PST1 and PST2) was repeated to confirm the predictions. An observation was made regarding differences observed between the predicted and the experimental values. Accordingly to the slight difference, it was considered that this model system was valid for the rest of the work. The optimal conditions were used, specifically to explain the mechanism of binding of the IC to the biomasses RBS, BST1, BST2, RPS, PST1, and PST2, by means of adsorption isotherms and kinetics modeling.

### 3.3. Kinetics Studies

**Figure 8:**
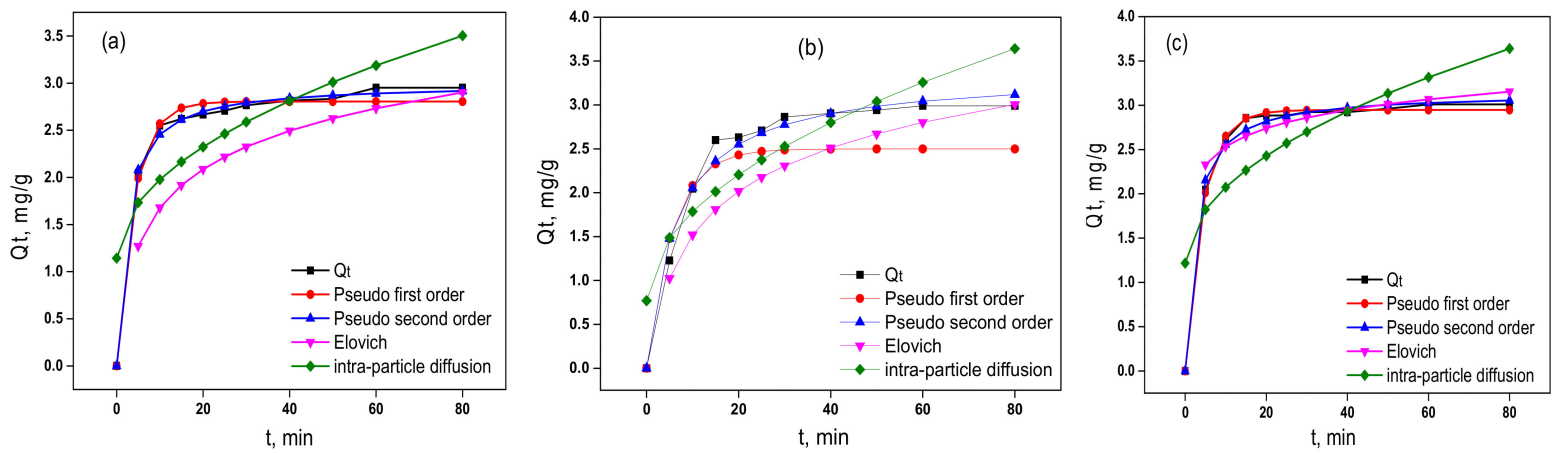
Graphs of nonlinear kinetics models for IC adsorption by RBS (**a**), BST1 (**b**) and BST2 (**c**).

**Figure 9:**
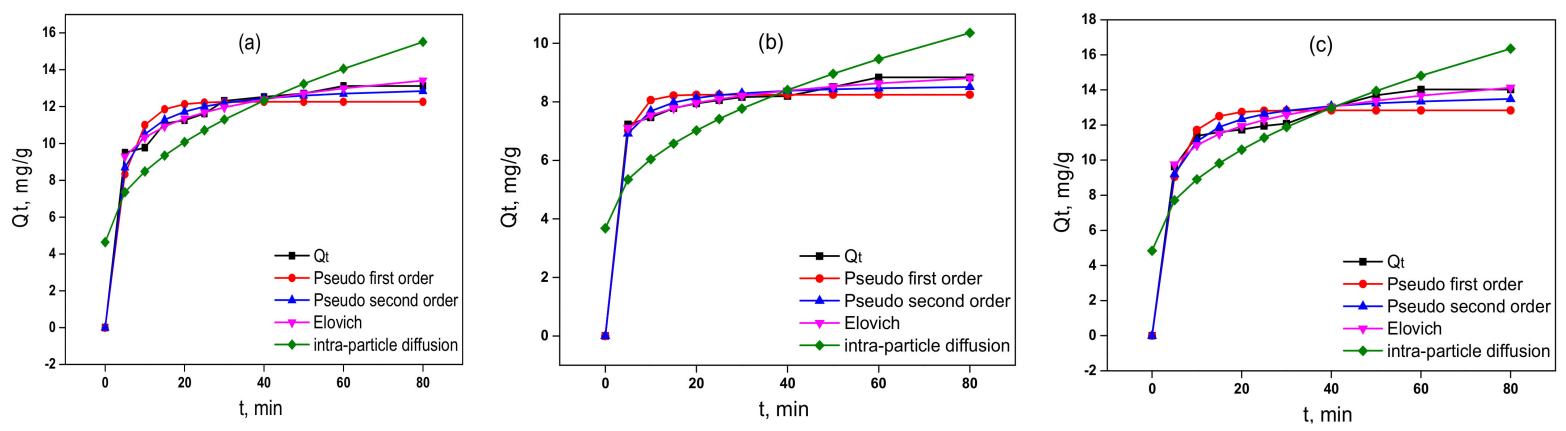
Graphs of nonlinear kinetics models of IC adsorption by RPS (**a**)**,** PST1 (**b**) and PST2 (**c**).

For the description of the adsorption process with a view to elucidating the mechanism of adsorption of IC on the different biomasses (RBS, BST1, BST2, RPS, PST1 and PST2), the contact time was varied between 5 and 60 min. The other parameters were fixed at their optimal values (Table 4). Kinetics models such as pseudo-first-order, pseudo-second-order, Elovich, and intra-particle diffusion models have been studied. Figure 8 and Figure 9 illustrate nonlinear plots. Table 5 and Table 6 show the values of the calculated constants of all the parameters obtained from the nonlinear regression forms.

The results in Table 5 and Table 6 show that the coefficients of determination of all the models are close to unity. These nonlinear models show that there is competition between the chemical and physical types of interactions during the adsorption of IC on our different materials. Based on the low values of χ^2^, the Elovich model better describes the adsorption kinetics of IC on the biomaterials RPS, PST1 and PST2; the pseudo-second-order model better describes the adsorption kinetics of IC on the biomaterials RBS, BST1 and BST2. These results are confirmed by the low values of the error functions REQM, HYBRID, SEA, and ERM. Elovich’s model assumes that the surface is energetically heterogeneous [84]. However, the pseudo-second-order model can also be considered as an alternative option to define the kinetics processes involved in this adsorption phenomenon, since it describes similar interactions at the surface level [85]. Low values of the desorption rate constant β around 0.650 mg/g.min obtained for biomaterials from RPS, PST1 and PST2 were observed, and such a trend highlights the multiplicity of sites available for sorption [86].

### 3.4. Isotherm Studies

**Figure 10:**
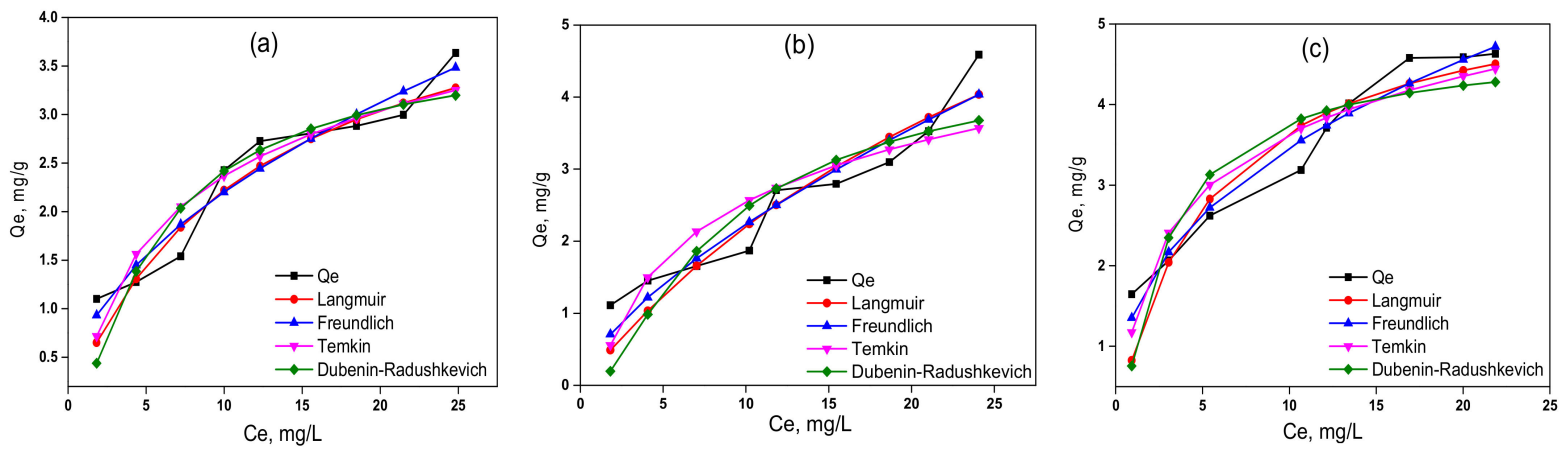
Adsorption isotherms of IC on (**a**) RBS, (**b**) BST1 and (**c**) BST2.

**Figure 11:**
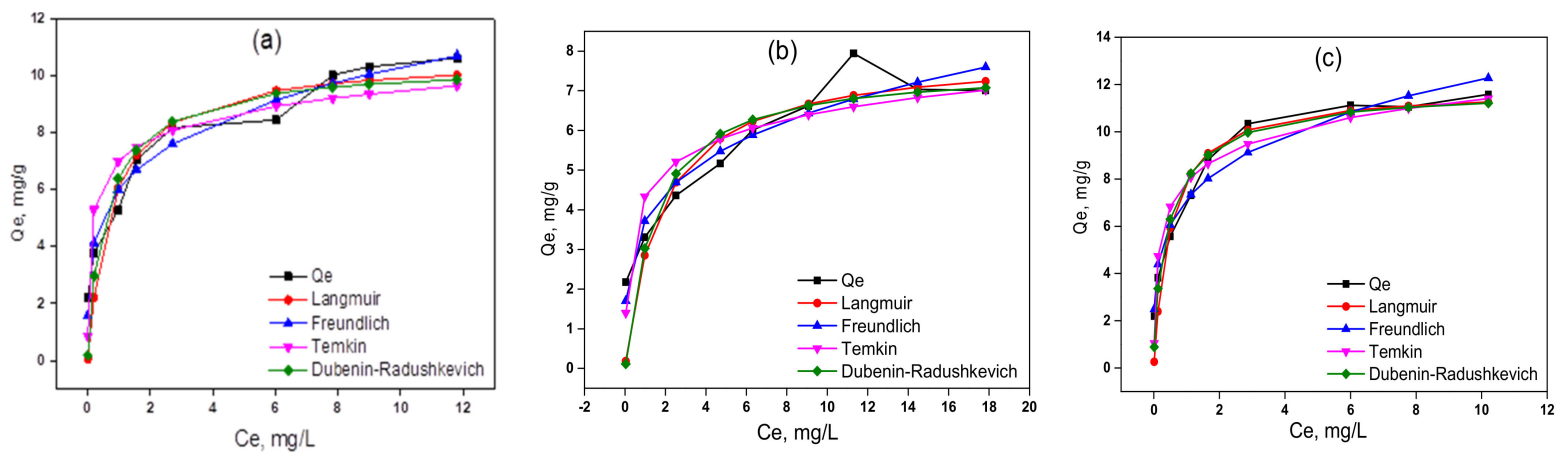
Adsorption isotherms of IC on (**a**) RPS, (**b**) PST1, and (**c**) PST2.

The amounts of IC in the liquid and solid phases, as well as the influence of the treatment of different materials on their retention, were elucidated on the basis of Langmuir, Freundlich, and Dubinin–Radushkevich (D–R) isotherms modeling. The uptake of IC onto BS samples (RBS, BST1 and BST2) and PS samples (RPS, PST1 and PST2) is plotted in Figure 10 and Figure 11, respectively, and the resulting data are listed in Table 7 and Table 8. It emerges from the tables that the low values of χ^2^ and the high values of the coefficient of determination R^2^, close to unity show that the best models used to describe the adsorption isotherm of the IC are those of Freundlich for all the studied biomaterials. This is confirmed by the low values of their REQM, HYBRID, SEA, and ERM error functions. Using these different errors, we can conclude that this isotherm is more suitable for the adsorption of IC. This model indicates a multilayer adsorption with heterogeneous adsorption sites and different energies [57]. Values of n greater than 1 for the Freundlich model showed the good affinity of the pollutant with the biomaterials and the adsorption process occurred on heterogeneous surfaces, characterizing adsorption at localized sites [57]. In addition, the more this value 1/n tends towards one, the greater the affinity. These results are in agreement with those of the kinetics studies.

The amounts of IC in the liquid and solid phases were also elucidated on the basis of Redlich–Peterson, Hill, Kaln, and Toth isotherm modeling. The uptake of IC onto BS samples (RBS, BST1, and BST2) and PS samples (RPS, PST1, and PST2) is plotted in Figures S13 and S14, respectively, and the resulting data are listed in Tables S12 and S13. It appears from these tables that the values of the coefficient of determination for all the isotherm models are close to unity (>0.9). In view of the high values of the coefficient of determination R^2^ and the low values of χ^2^, the Redlich–Peterson model is the most appropriate to describe the adsorption isotherm of IC. This is confirmed by the low values of the REQM, HYBRID, SEA and ERM error functions. The Redlich–Peterson isotherm argues for adsorption not following an ideal monolayer on a heterogeneous surface. The values of the heterogeneity parameter β of the Redlich–Peterson model are different from 1. These values (β less than 1) show that the model cannot be reduced to a monolayer adsorption, but rather to so-called heterogeneous system. These results are consistent with those obtained from two-parameter adsorption isotherm data, and kinetics studies, suggesting that the adsorption process occurred on heterogeneous surfaces. Other studies on the uptake of IC using others absorbent materials have been reported in the existing literature. In comparison with the other adsorbents, regarding the maximum adsorption capacities of IC of the studied adsorbents, the PST2 sample was found to be superior to some adsorbents (Table 9).

### 3.5. Desorption Studies

**Figure 12:**
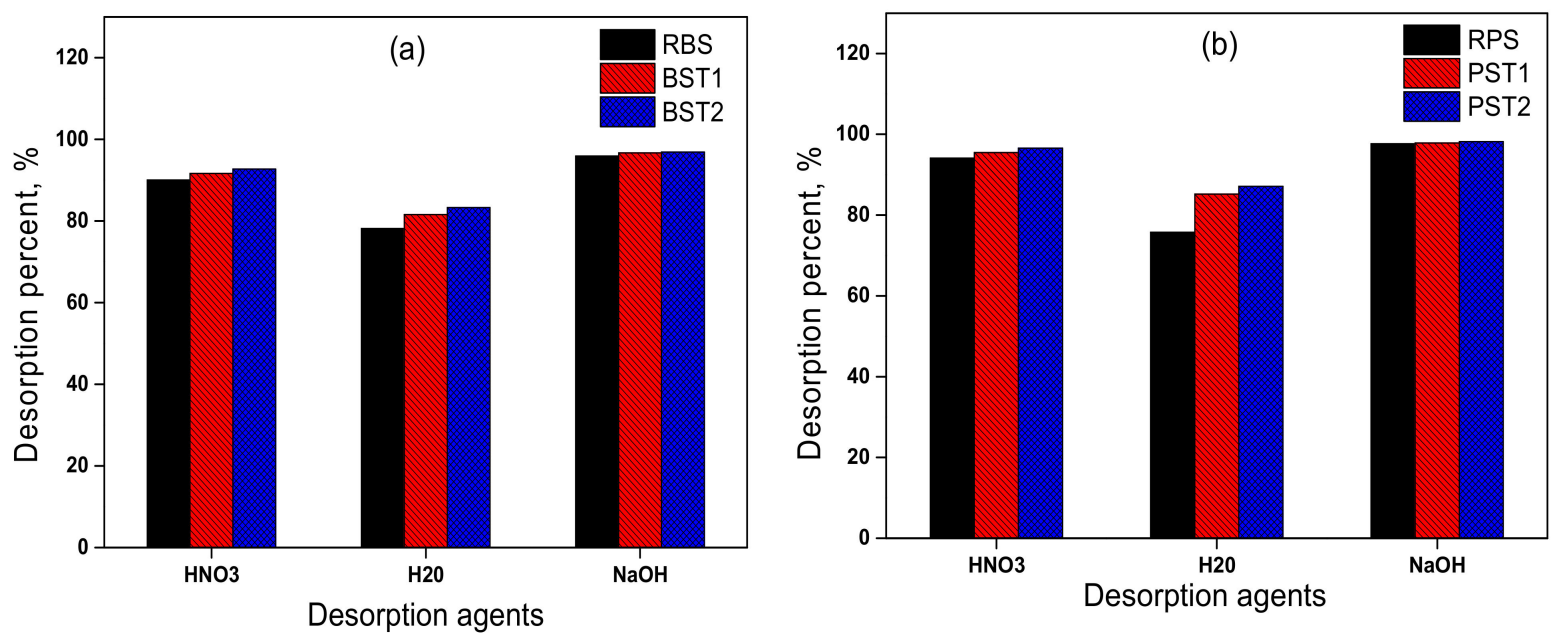
The desorption efficiency in three different media. Percent desorption of IC adsorbed onto biosorbents RBS, BST1, BST2 (**a**) and RPS, PST1, PST2 (**b**).

The possibility of reusing the different biosorbents after the adsorption of IC in the solution has been investigated through desorption studies. In order to consider the regeneration possibility of the adsorbent materials, the desorption studies were performed in three different media (acid, neutral and basic). Figure 12 shows the desorption efficiency of the tested regeneration agents. The basic medium was found to be the best desorbing reagent with efficiency of approximately 96%. Overall, the three studied media used allowed a good desorption of the IC with recorded desorption percentages greater than 70% for all the absorbent materials used. This could be explained by the fact that the IC molecules initially absorbed on adsorbent materials are retained by very low–energy interactions. To this end, the action of the strong acid (HNO_3_) or the strong base (NaOH) would certainly contribute to breaking the bonds initially formed between the chemical functions of IC in the solution and those of the adsorbent materials involved. This could lead to an ion exchange phenomenon between the Na^+^, OH^−^, H^+^, and NO_3_^−^ ions and the charged IC molecules in the solution depending on the pH of the acidic or basic desorbing solution. Considering the previous discussion,, there could be a form of competition between IC molecules and the Na^+^, OH^−^, H^+^ and NO_3_^−^ ions in the solution for the positively and negatively charged active sites of the absorbing materials. This could promote the rapid desorption of the initially adsorbed IC molecules.

## 4. Conclusions

The response surface methodology was used as a simple approach to study the effects of parameters such as the initial concentration of the water pollutant Indigo Carmine (IC), adsorbent mass, equilibrium time, and solution pH on the IC adsorption capacity of different lignocellulosic substrates extracted from waste biomasses from bean and pistachio pods. The physico-chemical characterizations carried out on the different absorbent materials (raw and chemically treated) highlighted the good absorbent properties of these biomaterials. These analyses revealed large specific surfaces and porosities for these materials, a semi-crystalline structure and materials rich in carbon, oxygen, and hydrogen elements and hydroxyl, carbonyl, and carboxyl chemical functions (coming from cellulose, lignin, and hemicellulose which are abundant in these biological materials), all essential for interactions with IC molecules during sorption processes. The optimal conditions were obtained for 50 mg/L of IC solution for the different biomaterials RBS, BST1, BST2, RPS, PST1, and PST2. The following optimal values were recorded with regard to the absorption of IC in aqueous solutions: dosage of adsorbent (50 mg), pH of the solution (2), and different stirring times depending on the sorbent involved, namely: 5 min for RBS, 38.42 min for BST1, 42.54 min for PST1 and 60 min for the biomaterials BST2, RPS, and PST2. These optimal conditions resulted in the removal of 1.81, 2.08, 3.56, 7.42, 8.95, and 15.35 mg/g respectively for the biomaterials RBS, BST1, BST2, RPS, PST1, and PST2. The parameters studied according to the analysis of the response surface all proved to have significant effects on the elimination of IC. Comparing the effects of the IC concentration and adsorbent mass, the effect of the IC concentration was found to have the greatest impact on the amount adsorbed. PST2 showed well-developed porosity and high adsorption capacity. The adsorption process was fast and followed a pseudo-second-order model for RBS, BST1, BST2 biomaterials and an Elovich model for the RPS, PST1, and PST2 biomaterials. From the experimental data, the nonlinear two-parameter Freundlich isotherm model, and the nonlinear three-parameter Redlich–Peterson isotherm model, fitted well with the biomaterials. This study showed that CCD-based RSM is an appropriate method to optimize the operating conditions and maximize the IC removal efficiency in aqueous media. Adsorption tests indicated that adsorbents derived from bean and pistachio pod residues pretreated with CH_4_N_2_O + HCl + (NH_4_)_2_S_2_O_8_ had good adsorption capacity for IC in aqueous solutions. The materials used in this work represent abundant, renewable, inexpensive biological matrices with good physico-chemical and absorbent properties that can be used for the treatment of aqueous effluents loaded with dyes. Desorption with a solution of nitric acid or sodium hydroxide made it possible to recover a maximum amount of IC in the range of 85 to 95%, depending on the absorbent material involved. However for the application of these materials for the decoloration of industrial effluents containing indigo carmine, it would be judicious to initially undertake studies of the selectivity of these biosorbents prepared with respect to the adsorption of indigo carmine in the presence of interferent species (heavy metals, pesticides, other dye molecules, etc.). Another aspect which can be addressed in future investigations is the study of the thermodynamics associated with the involved sorption processes including the determination of the related thermodynamic parameters. The exploitation on an industrial scale of the results of this study should be addressed, specially taking into account that these sorption processes implemented at the laboratory scale may differ from the processes fully industrially implemented, where the appropriate machines should be efficiently used.

## Figures and Tables

**Table 1:** Coded and experimental variables.

Experimental Variables	Units	Coded Variables	Variables Levels		
			−1	0	+1
Concentration	mg/L	A	5	27.5	50
pH	/	B	2	5	8
Time	min	C	5	32.5	60
Mass	mg	D	50	75	100

**Table 2:** Nonlinear forms of kinetics and isotherms models.

Models	Nonlinear Forms	References
Kinetics models		
Pseudo-first-order	$q_{t}=q_{e}(1-e^{-k_{1}t}$)	[57]
Pseudo-second-order	$q_{t}=\frac{k_{2}q_{e}^{2}t}{1+k_{2}q_{e}t}$	[57]
Elovich	$q_{t}=\frac{\ln\left(\alpha \beta \right)}{\beta }+\frac{\ln\left(t\right)}{\beta }$	[47,53]
Interparticle diffusion	qt=Kidt12+C	[58,59]
Two-parameter isotherms		
Langmuir	$Q_{e}=\frac{Q_{m}K_{L}C_{e}}{1+K_{L}C_{e}}$	[58,60]
Freundlich	$Q_{e}=K_{F}{\mathrm{Ce}}^{1/n}$	[58,60]
Dubinin–Radushkevich	$Q_{e}=Q_{m}\exp\left(-K^{\prime }\varepsilon^{2}\right)$ $\varepsilon =\mathrm{RTLn}\left(1+\frac{1}{C_{e}}\right)$ $E={\left(2K_{D}\right)}^{-1/2}$	[57]
Temken	$Q_{e}=\frac{RT}{Q}ln(A_{T}C_{e})$	[46,60]
Three-parameter isotherms		
Redlich–Peterson	$Q_{e}=\frac{AC_{e}}{1+BC_{e}^{\beta }}$	[61]
Toth	$Q_{e}=\frac{Q_{m}C_{e}}{{\left(K_{T}+C_{e}^{n}\right)}^{\frac{1}{n}}}$	[62]
Hill	$Q_{e}=\frac{Q_{SH}C_{e}^{nH}}{K_{D}+C_{e}^{nH}}$	[62]
Kahn	$Q_{e}=\frac{Q_{max}b_{K}C_{e}}{\left(1+b_{K}C_{e}\right)a_{k}}$	[62]

**Table 3:** Specific surfaces and pore volumes of studied materials.

Sample	Surface Area (m^2^/g)	Pore Volume (cm^3^/g)	Maximum Micropore Radius (nm)	Micropore Volume (cm^3^/g)	Micropore Surface (m^2^/g)
RBS	101.40	0.17	0.6007	0.011	50.050
BST1	139.69	0.21	1.033	0.089	60.627
BST2	151.13	0.23	1.4236	0.132	61.497
RPS	292.50	0.33	0.9798	0.033	60.799
PST1	331.11	0.49	1.3995	0.089	72.87
PST2	362.59	0.78	1.4592	0.168	85.07

**Table 4:** Validation of the experimental model of IC adsorption by the different biomasses (RBS, BST1, BST2, RPS, PST1, and PST2).

Biomasses	Variables Factors		Adsorbed Quantity (mg/g)		
			Experimental Values	Predicted Values	Residue
RBS	A	50	1.81	1.97	0.16
	B	2			
	C	5			
	D	50			
BST1	A	50	2.05	2.33	0.28
	B	2			
	C	38.42			
	D	50.16			
BST2	A	50	3.56	3.63	0.07
	B	2			
	C	60			
	D	50			
RPS	A	50	7.42	8.98	1.56
	B	2			
	C	60			
	D	50			
PST1	A	50	8.95	10.12	1.17
	B	2			
	C	42.54			
	D	50			
PST2	A	50	15.35	15.49	0.14
	B	2			
	C	60			
	D	50			

**Table 5:** Values of the constants of the nonlinear kinetics models for the biomasses RBS, BST1 and BST2.

Models	Constants	Value	R^2^	χ^2^	REQM	HYBRID	ERM	SEA
IC/RBS								
Pseudo first order	Q_e_ (mg/g)	2.804	0.999	0.003	0.087	0.014	0	0.010
	K_1_ (1/min)	0.248						
Pseudo second order	Q_e_ (mg/g)	2.996	0.999	0.001	0.044	0.007	0	0
	K_2_ (g/min.mg)	0.151						
Elovich	α (mg/g.min)	1.021	0.964	0.105	0.507	0.954	1.574	4.658
	β (mg/g.min)	1.7						
Intraparticle diffusion	K_p_ (mg/g.min^0.5^)	0.264	0.997	0.094	0.481	0.859	2.5866 × 10^−6^	7.65632 × 10^−6^
	C (mg/g)	1.143						
IC/BST1								
Pseudo first order	Q_e_ (mg/g)	2.498	0.899	0.286	0.822	1.304	0	0.078
	K_1_ (1/min)	0.179						
Pseudo second order	Q_e_ (mg/g)	3.367	0.997	0.006	0.119	0.055	0.018	0.052
	K_2_ (g/min.mg)	0.046						
Elovich	α (mg/g.min)	0.6	0.971	0.084	0.445	0.765	1.426	4.062
	β (mg/g.min)	1.4						
Intraparticle diffusion	K_p_ (mg/g.min^0.5^)	0.321	0.997	0.077	0.425	0.699	0	0
	C (mg/g)	0.769						
IC/BST2								
Pseudo first order	Q_e_ (mg/g)	2.946	0.999	0.001	0.061	0.014	0.002	0.006
	K_1_ (1/min)	0.229						
Pseudo second order	Q_e_ (mg/g)	3.142	0.999	0.001	0.038	0.003	0	0.006
	K_2_ (g/min.mg)	0.139						
Elovich	α (mg/g.min)	148.743	0.997	0.006	0.129	0.059	0	0
	β (mg/g.min)	3.362						
Intraparticle diffusion	K_p_ (mg/g.min^0.5^)	0.27	0.996	0.108	0.525	0.982	0	0
	C (mg/g)	1.217						

**Table 6:** Values of the constants of the nonlinear kinetics models for the biomasses RPS, PST1 and PST2.

Models	Constants	Value	R^2^	χ^2^	REQM	HYBRID	ERM	SEA
IC/RPS								
Pseudo first order	Q_e_ (mg/g)	12.259	0.996	0.054	0.760	0.247	0	0.212
	K_1_ (1/min)	0.227						
Pseudo second order	Q_e_ (mg/g)	13.257	0.998	0.016	0.412	0.145	0.052	0.004
	K_2_ (g/min.mg)	0.029						
Elovich	α (mg/g.min)	151.352	0.999	0.005	0.241	0.049	0	0
	β (mg/g.min)	0.671						
Intraparticle diffusion	K_p_ (mg/g.min^0.5^)	1.216	0.979	0.350	1.929	3.179	0	0
	C (mg/g)	4.637						
IC/PST1								
Pseudo first order	Q_e_ (mg/g)	8.245	0.998	0.018	0.367	0.083	0	0.017
	K_1_ (1/min)	0.381						
Pseudo second order	Q_e_ (mg/g)	8.638	0.999	0.007	0.222	0.061	0.001	0.007
	K_2_ (g/min.mg)	0.093						
Elovich	α (mg/g.min)	13735.063	0.999	0.001	0.094	0.011	0.002	0.016
	β (mg/g.min)	0.635						
Intraparticle diffusion	K_p_ (mg/g.min^0.5^)	0.746	0.981	0.308	1.506	2.801	0	0
	C (mg/g)	3.681						
IC/PST2								
Pseudo first order	Q_e_ (mg/g)	12.841	0.996	0.058	0.808	0.265	0	0.110
	K_1_ (1/min)	0.244						
Pseudo second order	Q_e_ (mg/g)	13.919	0.998	0.022	0.494	0.198	0.003	0.041
	K_2_ (g/min.mg)	0.028						
Elovich	α (mg/g.min)	152.928	0.999	0.008	0.293	0.070	0	0
	β (mg/g.min)	0.634						
Intraparticle diffusion	K_p_ (mg/g.min^0.5^)	1.288	0.979	0.359	2.005	3.264	0	0
	C (mg/g)	4.835						

**Table 7:** Data from the Langmuir, Freundlich, and D–K–R isotherms for the sorption of IC by RBS, BST1, and BST2 samples.

Model	Constant	Value	R^2^	χ^2^	REQM	HYBRID	ERM	SEA
IC/RBS								
Langmuir	Q_m_ (mg/g)	4.814	0.990	0.025	0.278	0.363	0.156	0.234
	K_L_ (L/min)	0.086						
Freundlich	K_F_ (mg/L)	0.688	0.993	0.018	0.237	0.262	0.009	0.014
	n	1.980						
Temkin	A_T_ (L/mg)	1.144	0.988	0.032	0.312	0.457	0	0
	ΔQ (J/mol)	2550.507						
Dubenin-Raduschkevich	Q_m_ (mg/g)	3.896	0.984	0.043	0.362	0.615	0.221	0.332
	K_id_ (L/mg)	0.001						
	E (kJ/mol)	22.360						
IC/BST1								
Langmuir	Q_m_ (mg/g)	9.866	0.982	0.055	0.424	0.789	0.403	0.643
	K_L_ (L/min)	0.029						
Freundlich	K_F_ (mg/L)	0.474	0.987	0.038	0.356	0.556	0.142	0.226
	N	1.485						
Temkin	A_T_ (L/mg)	0.888	0.968	0.095	0.557	1.363	0	0
	ΔQ (J/mol)	2125.651						
Dubenin-Raduschkevich	Q_m_ (mg/g)	4.964	0.964	0.109	0.598	1.570	0.519	0.829
	K_id_ (L/mg)	0.001						
	E (kJ/mol)	22.360						
IC/BST2								
Langmuir	Q_m_ (mg/g)	5.601	0.990	0.038	0.413	0.550	0.233	0.506
	K_L_ (L/min)	0.188						
Freundlich	K_F_ (mg/L)	1.398	0.996	0.011	0.228	0.168	0.029	0.064
	n	2.536						
Temkin	A_T_ (L/mg)	3.387	0.991	0.033	0.383	0.472	0	0
	ΔQ (J/mol)	2399.951						
Durbenin-Raduschkevich	Q_m_ (mg/g)	4.788	0.983	0.065	0.537	0.929	0.185	0.401
	K_id_ (L/mg)	0.001						
	E (kJ/mol)	22.360						

**Table 8:** Data from the Langmuir, Freundlich, and D–K–R isotherms for the sorption of IC by RPS, PST1, and PST2 samples.

Model	Constant	Value	R^2^	χ^2^	REQM	HYBRID	ERM	SEA
IC/RPS								
Langmuir	Q_m_ (mg/g)	10.654	0.984	0.143	1.161	2.045	0.635	2.933
	K_L_ (L/min)	1.347						
Freundlich	K_F_ (mg/L)	6.039	0.996	0.032	0.556	0.469	0.051	0.237
	n	4.310						
Temken	A_T_(L/mg)	753.037	0.982	0.152	1.196	2.172	0	0
	ΔQ(J/mol)	2334.670						
Durbenin-Raduschkevich	Q_m_(mg/g)	10.441	0.986	0.122	1.075	1.755	0.434	2.002
	K_id_(L/mg)	0.0001						
	E (kJ/mol)	70.710						
IC/PST1								
Langmuir	Q_m_ (mg/g)	7.950	0.981	0.119	0.918	1.701	0.573	1.992
	K_L_ (L/min)	0.572						
Freundlich	K_F_(mg/L)	3.739	0.992	0.047	0.579	0.677	0.032	0.112
	n	4.063						
Temken	A_T_(L/mg)	110.626	0.985	0.094	0.816	1.343	0	0
	ΔQ(J/mol)	2677.737						
Durbenin–Raduschkevich	Q_m_(mg/g)	7.601	0.979	0.132	0.968	1.890	0.550	1.913
	K_id_(L/mg)	0.0002						
	E (kJ/mol)	50.000						
IC/PST2								
Langmuir	Q_m_ (mg/g)	11.800	0.990	0.098	1.002	1.400	0.512	2.575
	K_L_ (L/min)	2.034						
Freundlich	K_F_(mg/L)	7.138	0.994	0.049	0.711	0.704	0.055	0.280
	n	4.283						
Temken	A_T_ (L/mg)	181.799	0.992	0.074	0.876	1.068	0.000	0.000
	ΔQ (J/mol)	1634						
Durbenin-Raduschkevich	Q_m_ (mg/g)	11.832	0.994	0.051	0.724	0.731	0.194	0.976
	K_id_ (L/mg)	0.0001						
	E (kJ/mol)	70.710						

**Table 9:** Adsorption amounts of IC onto some adsorbent materials.

Absorbent Material	Adsorption Capacity (mg/g)	Absorption pH	Reference
Brazil nut shell	1.09	6.50	[87]
Rice husk ash	29.28	5.40	[88]
Bottom ash	0.00017	2	[89]
De-oiled soya	0.00038	2	[89]
Chitin	0.0058	5	[90]
Chitosan	0.072	5	[90]
Pistia stratiotes	41.2	5	[91]
Carbonaceous material (tea waste)	20	6	[92]
Dried cola nut shell activated with KOH	13	2	[93]
Acacia nilotica (babool) sawdust activated carbon	4.8	2	[94]
Cellulosic waste of citrus reticulate peels obtained formaldehyde	15	8.50	[95]
Carbonaceous material from pyrolysis of sewage sludge	93	6.50	[96]
Commercial activated carbon	79	2	[97]
*Moringa oleifera* seeds (nanosized adsorbent)	60.2	4	[98]
*Moringa oleifera* seeds (bulk adsorbent)	14	4	[98]
*Activated pomegranate peel*	158.73	2	[99]
Raw bean pods	1.81	2	This study
Bean pods treated with NaOH+HCl	2.05	2	This study
Bean pods treated with CH_4_N_2_O+HCl+(NH_4_)_2_S_2_O_8_	3.56	2	This study
Raw pistachio pods	7.42	2	This study
Pistachio pods treated with NaOH+HCl	8.95	2	This study
Pistachio pods treated with CH_4_N_2_O+HCl+(NH_4_)2S_2_O_8_	15.35	2	This study

## Data Availability

Not applicable.

## Supplementary Materials

The following supporting information can be downloaded at: https://www.mdpi.com/article/10.3390/polym14183776/s1, Figure S1. Point of zero charge graphs for bean pods (a) and pistachio pods (b): RBS and RPS (black curve), BST1 and PST1 (red curve) and BST2 and PST2 (blue curve). Figure S2. EDX spectra of RBS (a), BST2 (b), RPS (c) and PST2 (d). Figure S3. Pareto diagram for the amount of IC adsorbed by the RBS. Figure S4. Pareto diagram for the amount of IC adsorbed by BST1. Figure S5. Pareto diagram for the amount of IC adsorbed by BST2. Figure S6. Pareto diagram for the amount of IC adsorbed by the RPS. Figure S7. Pareto diagram for the amount of IC adsorbed by PST1. Figure S8. Pareto diagram for the amount of IC adsorbed by PST2. Figure S9. Surface responses for the amount of IC adsorbed by RBS as function of concentration and pH (a), concentration and mass (b), and of the effects of pH and the mass (c). Figure S10. Surface response for the amount of IC adsorbed by BST1 as a function of concentration and pH effects. Figure S11. Surface responses for the amount of IC adsorbed by the RPS as a function of the effects of concentration and pH (a), of the effects of concentration and time (b). Figure S12. Surface responses for the amount of IC adsorbed by PST1 as a function of concentration and pH effects (a), pH effects and mass (b). Figure S13. Adsorption isotherms of IC on (a) RBS, (b) BST1 and (c) BST2 materials. Figure S14. Adsorption isotherms of IC on (a) RPS, (b) PST1 and (c) PST2 materials. Table S1. Error functions and their equations. Table S2. Main diffraction peaks observed in bean pods. Table S3. Main diffraction peaks observed in pistachio pods. Table S4. Experimental design matrix for the removal of IC onto RBS, BST1 and BST2 materials using central composite design. Table S5. Experimental design matrix for the removal of IC onto RPS, PST1 and PST2 materials using central composite design. Table S6. Analysis of variance of the amount of IC adsorbed by RBS material. Table S7. Analysis of variance of the amount of IC adsorbed BST1 material. Table S8. Analysis of variance of the amount of IC adsorbed by BST2 material. Table S9. Analysis of variance of the amount of IC adsorbed by RPS material. Table S10. Analysis of variance of the amount of IC adsorbed by PST1 material. Table S11. Analysis of variance of the amount of IC adsorbed by PST2 material. Table S12. Data from the Redlich–Peterson, Hill, Kaln and Toth isotherms for the sorption of IC by RBS, BST1 and BST2 materials. Table S13. Data from the Redlich-Peterson, Hill, Kaln and Toth isotherms for the sorption of IC by RPS, PST1 and PST2 materials.
