# Supplementary material for: Lignocellulosic-Based Materials from Bean and Pistachio Pod Wastes for Dye-Contaminated Water Treatment: Optimization and Modeling of Indigo Carmine Sorption
Source: Polymers (Basel). 2022 Sep 9;14(18):3776. doi: 10.3390/polym14183776 (PMC9504809; doi:10.3390/polym14183776)
Supplement: Supplementary file 1 [file polymers-14-03776-s001.zip › polymers-1854151-supplementary.pdf]

Lignocellulosic-Based Materials from Bean and Pistachio Pod Wastes for Dye-Contaminated Water Treatment: Optimization and Modeling of Indigo Carmine Sorption

Gaël Ferdinand Kazé Nindjio [1], Rufis Fregue Tiegam Tagne [2], Sherman Lesly Zambou Jiokeng [1,3], Cyrille Ghislain Fotsop [4], Aurelien Bopda [1], Giscard Doungmo [5], Ranil Clément Tonleu Temgoua [1,6], Ingo Doench [7,8,9], Estella Tamungang Njoyim [1,10], Arnaud Kamdem Tamo [7,8,9,*], Anayancy Osorio-Madrazo [7,8,9,*], Ignas Kenfack Tonle [1,*]

[1] Research Unit of Noxious Chemistry and Environmental Engineering, Department of Chemistry, University of Dschang, P.O.Box. 67 Dschang, Cameroon
[2] Department of Paper Sciences and Bioenergy, University Institute of Wood Technology, University of Yaoundé I, Mbalmayo, P.O.Box. 306, Cameroon
[3] Institut für Anorganische Chemie und Strukturchemie, Heinrich-Heine-Universität Düsseldorf, 40204 Düsseldorf, Germany
[4] Otto-von-Guericke-University Magdeburg, Chemical Institute Industrial Chemistry, Universitatsplatz 2, 39106 Magdeburg, Germany
[5] Institut für Anorganische Chemie, Christian-Albrechts-Universität zu Kiel, Max-Eyth-Str. 2, 24118, Kiel, Germany
[6] Higher Teacher Training College, University of Yaoundé 1, Yaoundé P.O. Box 47, Cameroon
[7] Laboratory for Bioinspired Materials BMBT, Institute of Microsystems Engineering IMTEK-Sensors, University of Freiburg, 79110 Freiburg, Germany
[8] Freiburg Center for Interactive Materials and Bioinspired Technologies FIT, University of Freiburg, 79110 Freiburg, Germany;
[9] Freiburg Materials Research Center FMF, University of Freiburg, 79104 Freiburg, Germany
[10] Department of Chemistry, Higher Teacher Training College, University of Bamenda, Bambili P.O.Box. 39, Cameroon

Correspondence: arnaud.kamdem@imtek.uni-freiburg.de (A.K.T.); anayancy.osorio@imtek.uni-freiburg.de (A.O.-M.); ignas.tonle@univ-dschang.org (I.K.T.); Tel.: +49-761-203-95096 (A.K.T.); +49-761-203-67363 (A.O.-M.); +237-696-141-545 (I.K.T.)

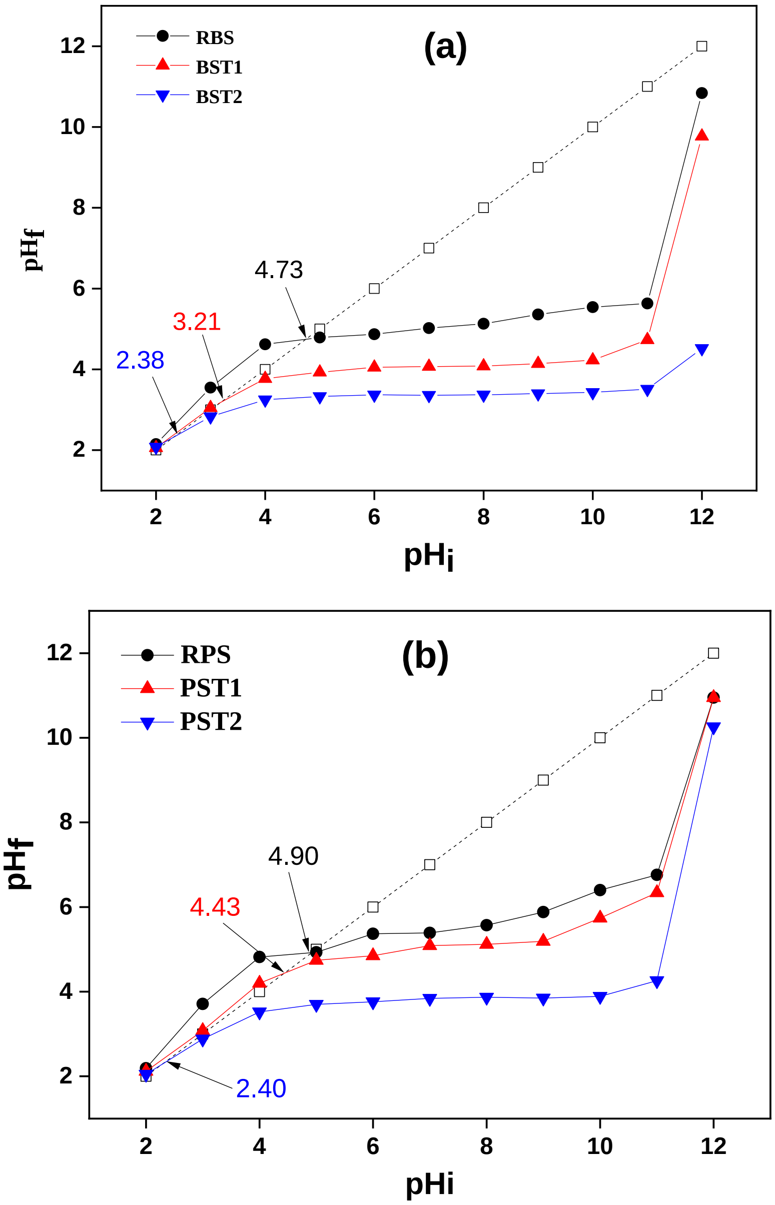

Figure S1. Point of zero charge graphs for bean pods **(a)** and pistachio pods **(b)**: RBS and RPS (black curve), BST1 and PST1 (red curve) and BST2 and PST2 (blue curve).

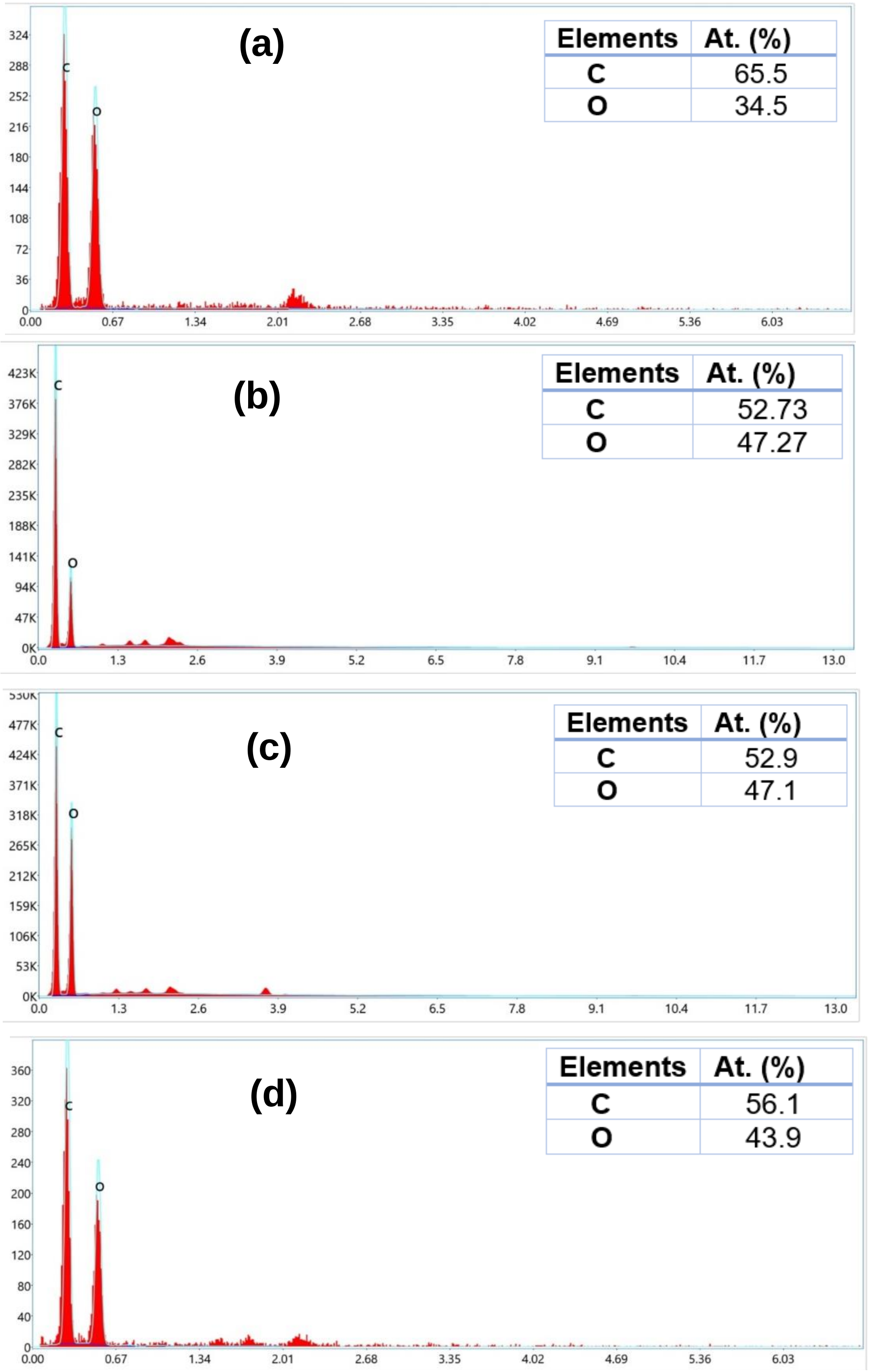

Figure S2. EDX spectra of RBS **(a)**, BST2 **(b),** RPS **(c)** and PST2 **(d)**.

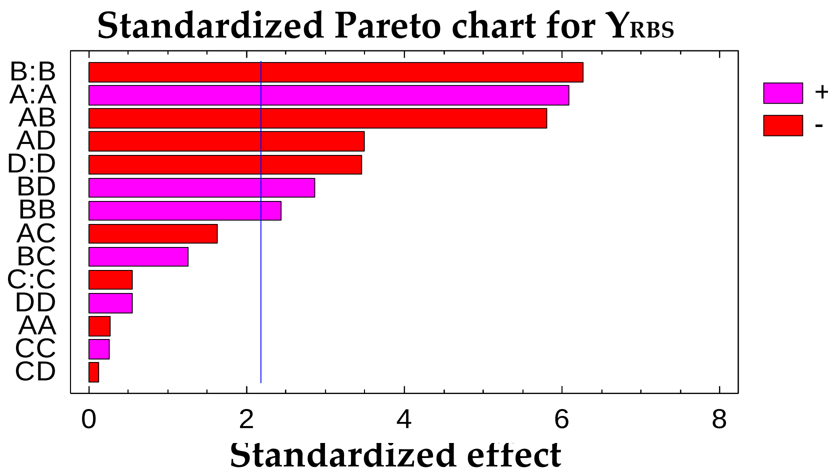

Figure S3. Pareto diagram for the amount of IC adsorbed by the RBS

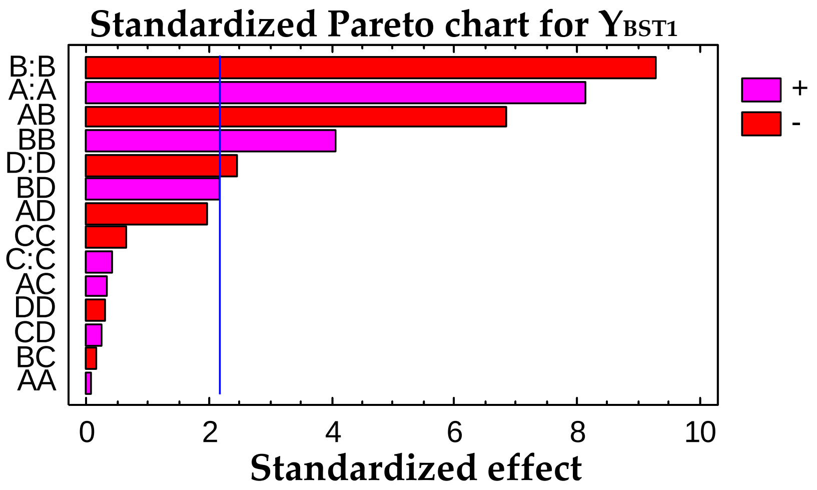

Figure S4. Pareto diagram for the amount of IC adsorbed by BST1

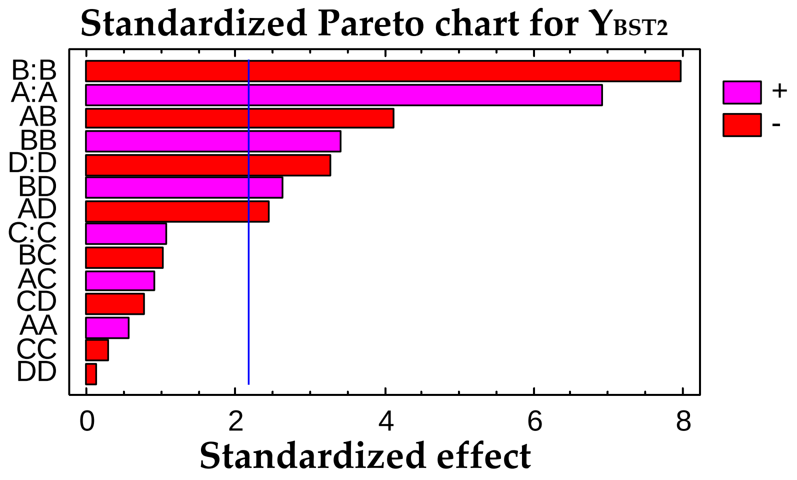

Figure S5. Pareto diagram for the amount of IC adsorbed by BST2.

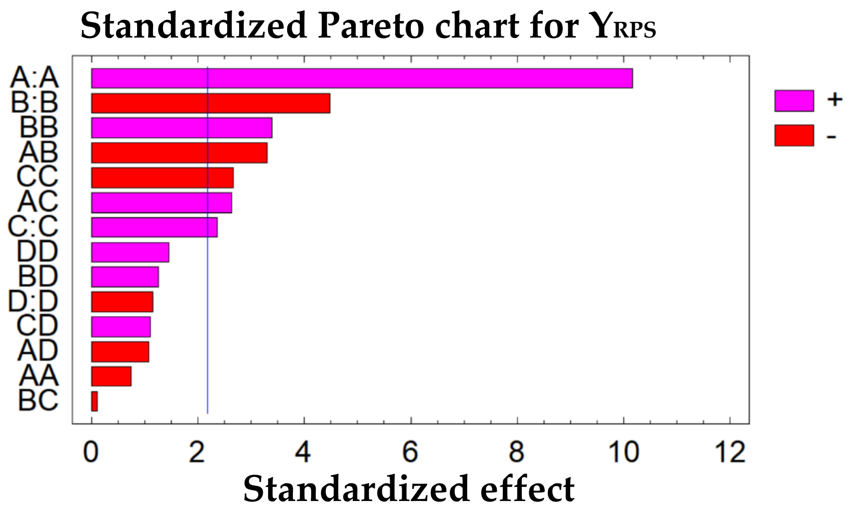

Figure S6. Pareto diagram for the amount of IC adsorbed by the RPS

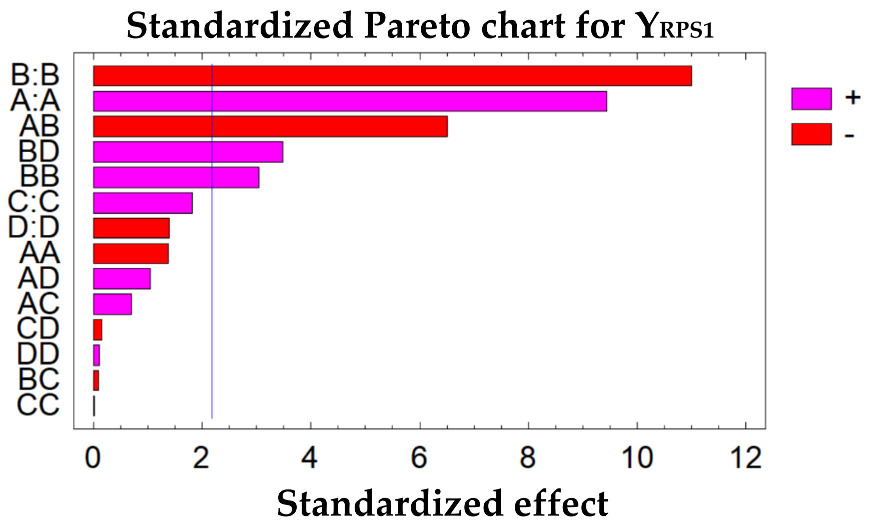

Figure S7. Pareto diagram for the amount of IC adsorbed by PST1.

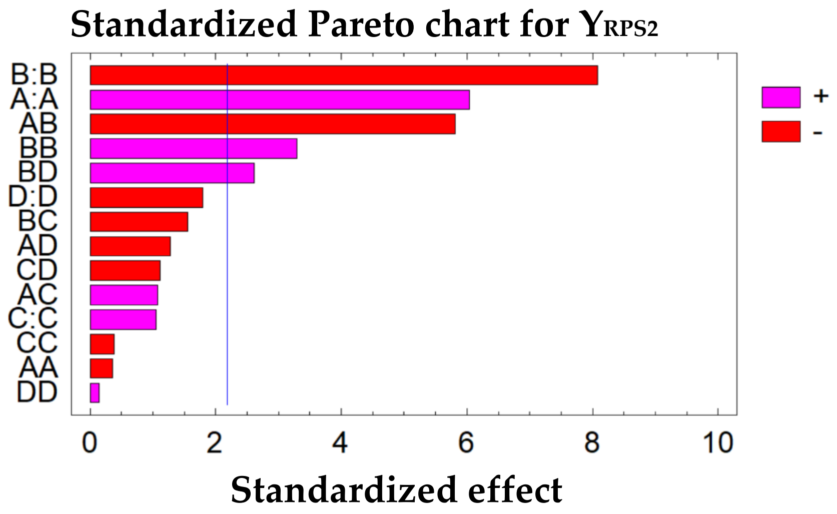

Figure S8. Pareto diagram for the amount of IC adsorbed by PST2.

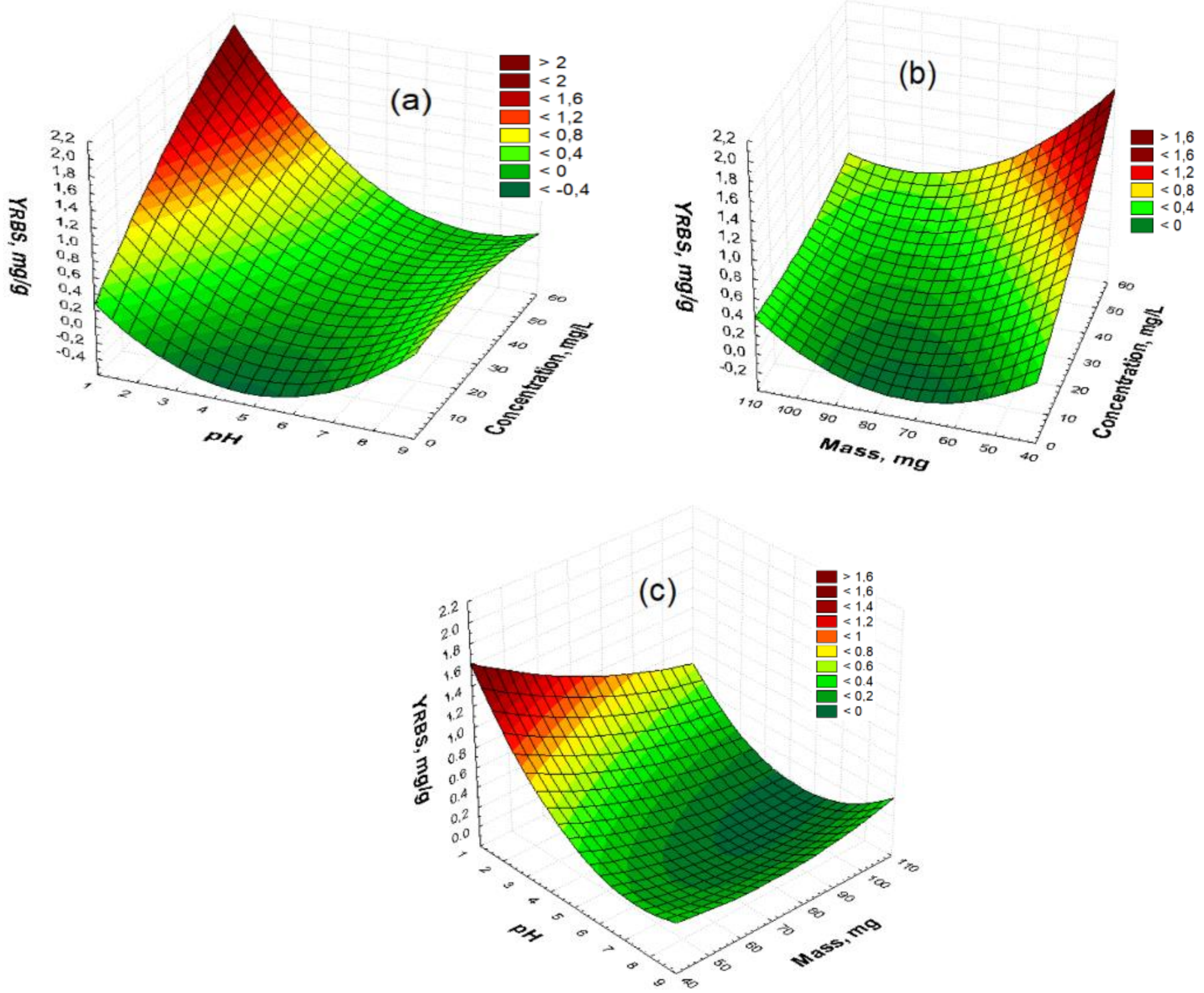

Figure S9. Surface responses for the amount of IC adsorbed by RBS as function of concentration and pH (a), concentration and mass (b), and of the effects of pH and the mass (c).

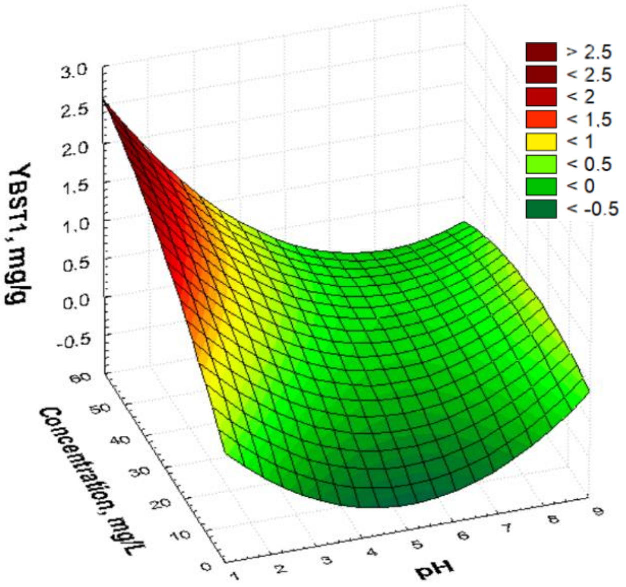

Figure S10. Surface response for the amount of IC adsorbed by BST1 as a function of concentration and pH effects.

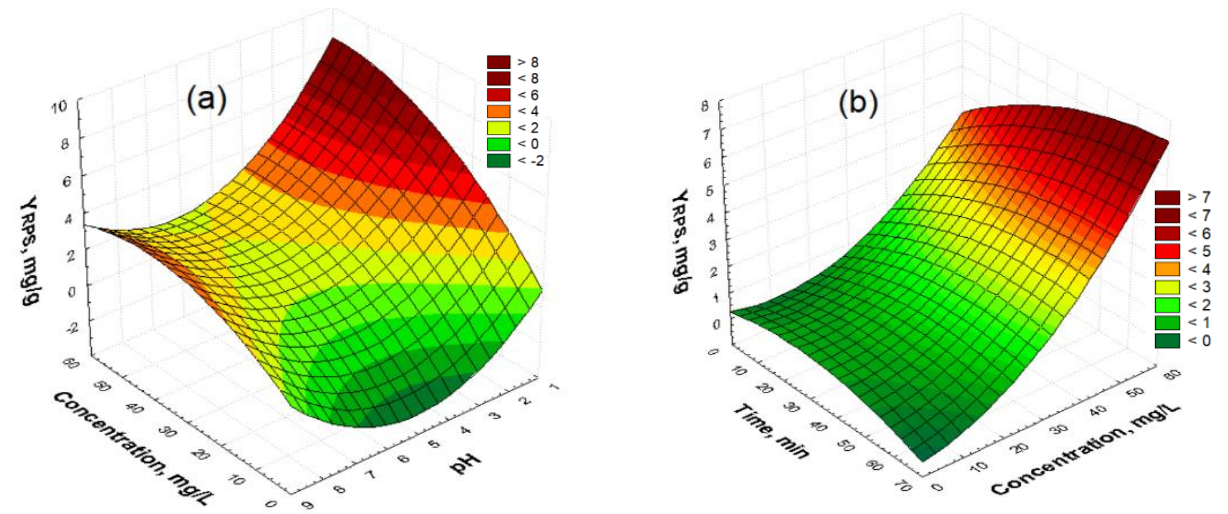

Figure S11. Surface responses for the amount of IC adsorbed by the RPS as a function of the effects of concentration and pH (a), of the effects of concentration and time (b).

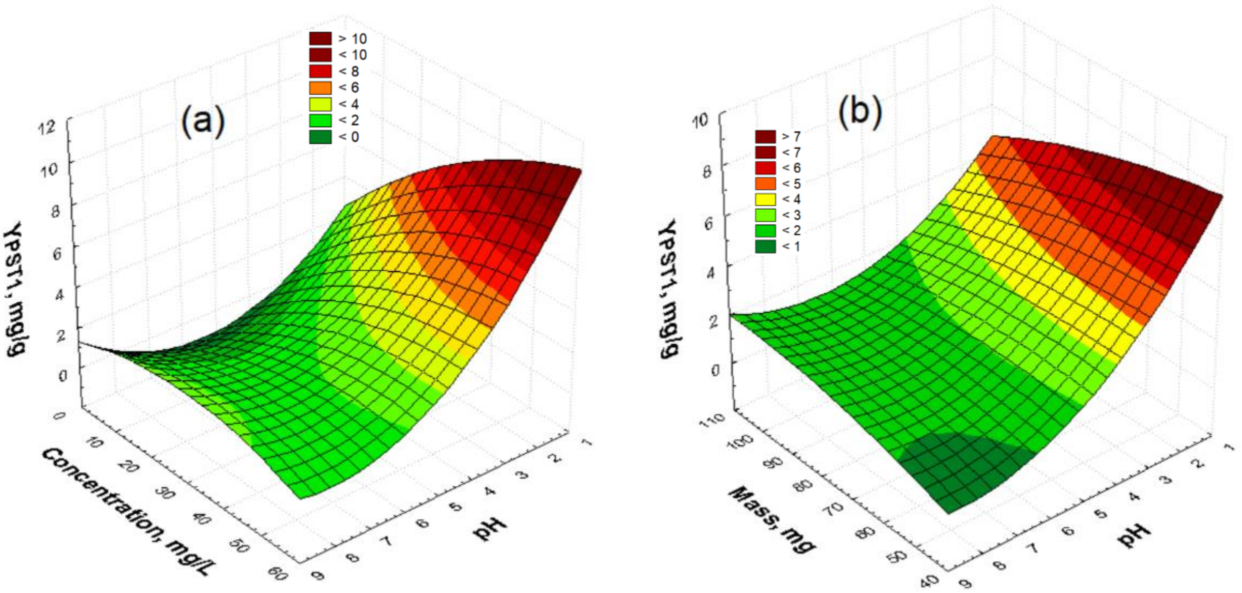

Figure S12. Surface responses for the amount of IC adsorbed by PST1 as a function of concentration and pH effects (a), pH effects and mass (b).

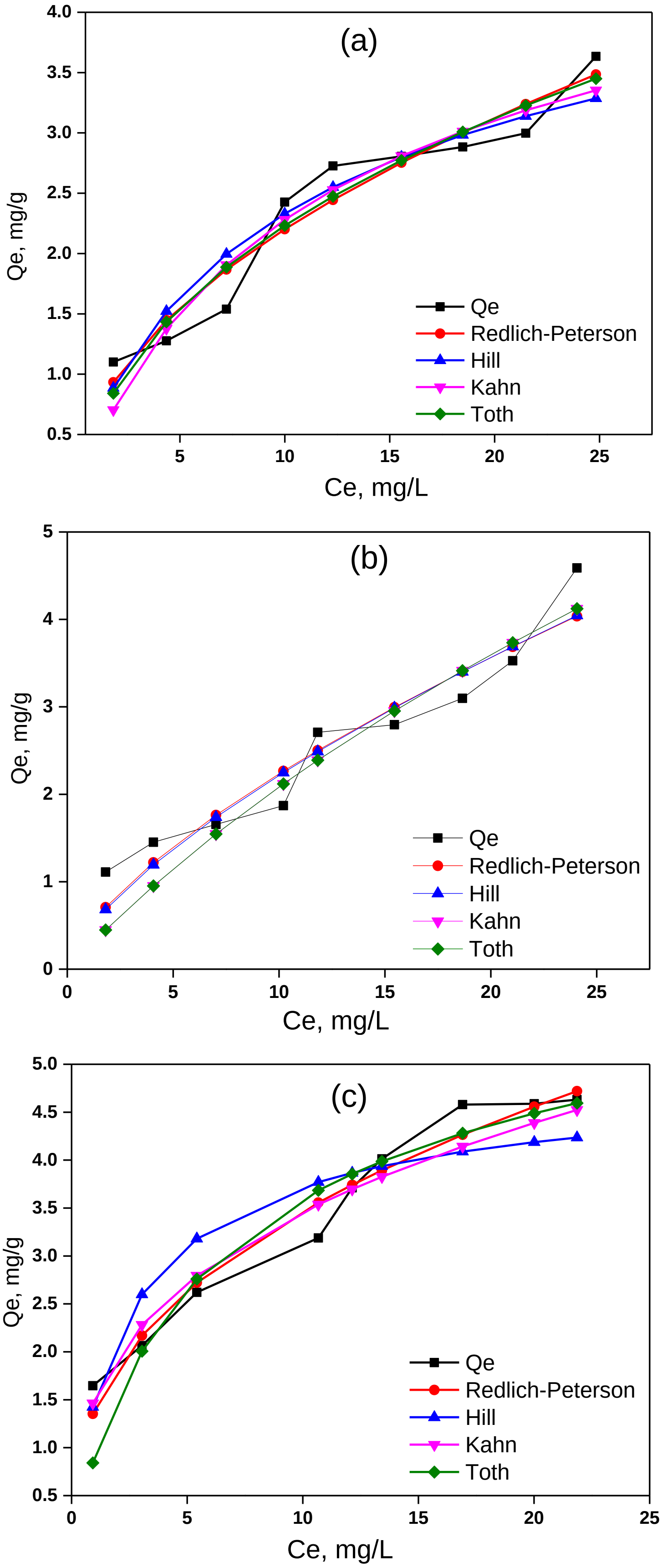

Figure S13. Adsorption isotherms of IC on (a) RBS, (b) BST1 and (c) BST2 materials.

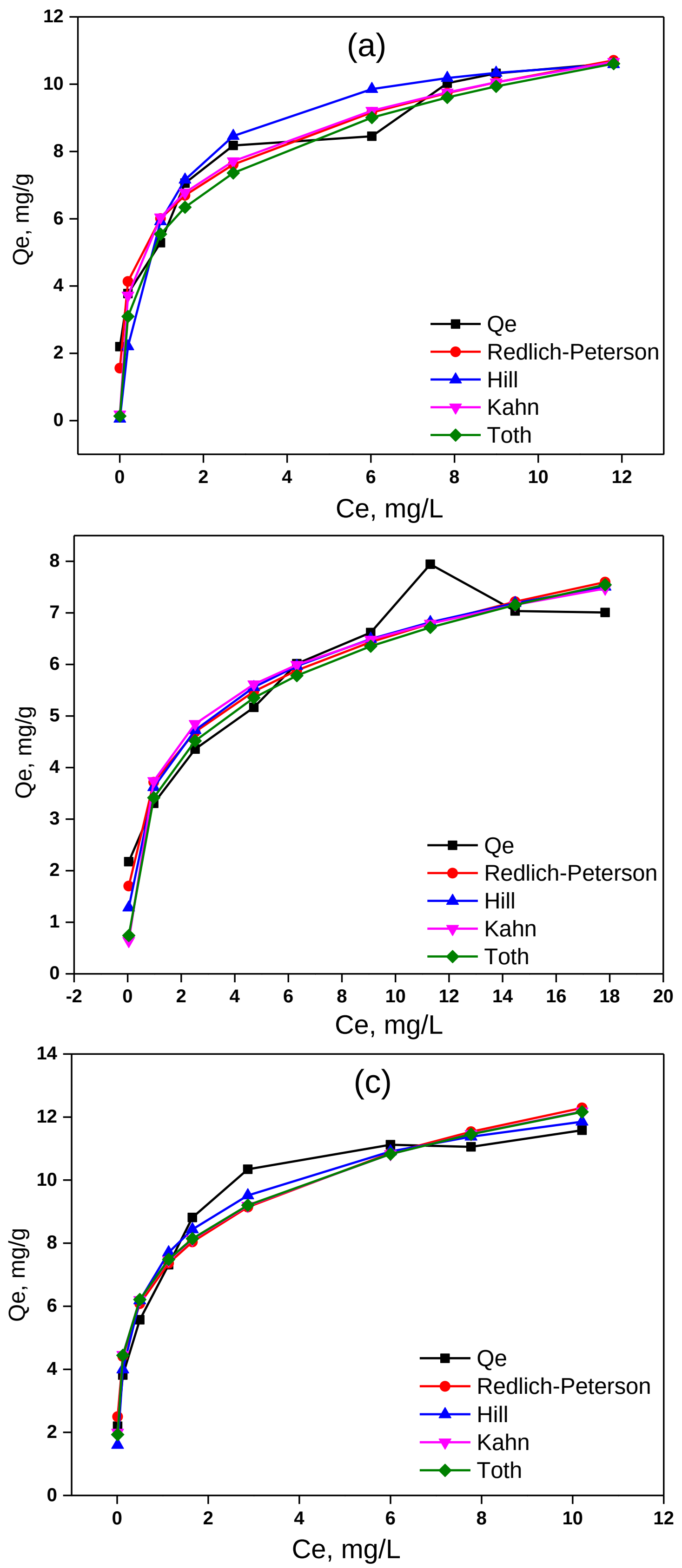

Figure S14. Adsorption isotherms of IC on (a) RPS, (b) PST1 and (c) PST2 materials.

Table S1. Error functions and their equations

Error functions	Abbreviations	Formulas
Residual Root Mean Square Error	RMSE	$\sqrt{\left(\frac{1}{n-1}\sum_{i=1}^{n}(q_{e,exp}-q_{e,cal})\right)^2}$
Average Relative Error	ARE	$\frac{100}{n}\sum_{i=1}^{n}\left\lvert\frac{q_{e,exp}-q_{e,cal}}{q_{e,exp}}\right\rvert_i$
Sun of absolute errors	EABS	$\sum_{i=1}^{n}\lvert q_{e,exp,i}-q_{e,cal,i}\rvert$
Hybrid fractional error function	HYBRID	$\frac{100}{n-p}\sum_{i=1}^{n}\left[\frac{(q_{e,exp}-q_{e,cal})^2}{q_{e,exp}}\right]$
Nonlinear chi-square test	χ^2	$\sum_{i=1}^{n}\frac{(q_{e,cal}-q_{e,exp})^2}{q_{e,exp}}$
Coefficient of determination	R^2	$\frac{(q_{e,exp}-\overline{q_{e,cal}})^2}{\sum(q_{e,exp}-\overline{q_{e,cal}})^2+(q_{e,exp}-q_{e,cal}}$

Table S2. Main diffraction peaks observed in bean pods

Samples	2θ	d	Amorphous phase (%)	Diffraction peaks (%)
RBS	21.46	4.1406	93.24	6.76
	34.54	2.5968		
BST1	22.08	4.0252	90.83	9.17
	34.54	2.5999		
BST2	21.37	4.1576	92.92	7.08
	34.81	2.5775		

Table S3. Main diffraction peaks observed in pistachio pods

Samples	2θ	d	Amorphous phase (%)	Diffraction peaks (%)
RPS	20.93	4.2450	92.37	7.63
	26.44	3.3706		
	42.46	2.1290		
	50.11	1.8204		
	54.92	1.6719		
	60.08	1.5401		
	68.17	1.3756		
PST1	21.55	4.1406	93.53	6.47
	26.62	3.3485		
	36.68	2.4503		
	50.11	1.8204		
	59.81	1.5463		
	67.82	1.3819		
PST2	20.93	4.2450	93.74	6.26
	26.44	3.3706		
	39.52	2.2801		
	40.41	2.2320		
	55.27	1.6620		
	68.09	1.3771		

Table S4. Experimental design matrix for the removal of IC onto RBS, BST1 and BST2 materials using central composite design.

Run	Factors				Q_{ads} (mg/g)								
					RBS			BST1			BST2		
No	A	B	C	D	E.v.	P.v.	R.	E.v.	P.v.	R.	E.v.	P.v.	R.
1	+1	-1	+1	+1	0.48	1.00	0.52	1.33	1.73	0.4	1.55	2.29	0.74
2	+1	-1	+1	-1	1.81	1.97	0.16	2.05	2.33	0.28	3.56	3.63	0.07
3	0	0	-1	0	0.09	0.43	0.34	0.09	0.34	0.25	0.16	0.53	0.37
4	0	0	0	0	0.07	0.23	0.16	0.08	0.28	0.2	0.22	0.43	0.21
5	-1	+1	+1	+1	0.27	0.76	0.49	0.03	0.49	0.46	0.08	0.63	0.55
6	-1	-1	-1	-1	0.15	0.63	0.48	0.19	0.70	0.51	0.67	1.22	0.55
7	0	+1	0	0	0.16	0.39	0.23	0.02	0.50	0.48	0.12	0.73	0.61
8	-1	0	0	0	0.05	0.05	0.00	0.06	0.05	-0.01	0.03	0.17	0.14
9	+1	+1	-1	+1	0.07	0.34	0.27	0.12	0.33	0.21	0.43	0.76	0.33
10	-1	-1	-1	+1	0.06	0.38	0.32	0.12	0.44	0.32	0.41	0.84	0.43
11	+1	+1	+1	-1	0.26	0.64	0.38	0.26	0.59	0.33	0.66	1.32	0.66
12	+1	-1	-1	-1	2.07	2.29	0.22	2.08	2.27	0.19	2.53	3.08	0.55
13	-1	+1	-1	-1	0.03	0.22	0.19	0.07	0.32	0.25	0.10	0.46	0.36
14	0	-1	0	0	0.59	0.97	0.38	1.28	1.35	0.07	1.60	1.87	0.27
15	0	0	0	+1	0.09	0.28	0.19	0.13	0.28	0.15	0.20	0.41	0.21
16	-1	+1	+1	-1	0.08	0.46	0.38	0.04	0.29	0.25	0.12	0.44	0.32
17	-1	-1	+1	+1	0.10	0.36	0.26	0.13	0.48	0.35	0.45	0.90	0.45
18	+1	+1	+1	+1	0.05	0.25	0.20	0.20	0.41	0.21	0.46	0.78	0.32
19	0	0	0	0	0.07	0.23	0.16	0.08	0.28	0.2	0.22	0.43	0.21
20	0	0	0	-1	0.19	0.61	0.42	0.12	0.51	0.39	0.20	0.87	0.67
21	-1	+1	-1	+1	0.01	0.53	0.52	0.03	0.47	0.44	0.07	0.87	0.8
22	-1	-1	+1	-1	0.20	0.63	0.43	0.26	0.70	0.44	0.73	1.50	0.77
23	0	0	0	0	0.073	0.23	0.16	0.08	0.28	0.2	0.22	0.43	0.21
24	0	0	+1	0	0.12	0.38	0.26	0.08	0.37	0.29	0.18	0.68	0.5
25	+1	-1	-1	+1	1.05	1.35	0.3	1.16	1.63	0.47	1.42	1.97	0.55
26	+1	0	0	0	0.01	0.62	0.61	0.25	0.80	0.55	0.43	1.17	0.74
27	+1	+1	-1	-1	0.30	0.72	0.42	0.19	0.56	0.37	0.66	1.08	0.42

E.v.: Experimental values, P.v.: Predicted values, R: Residuals

Table S5. Experimental design matrix for the removal of IC onto RPS, PST1 and PST2 materials using central composite design.

Run	Factors				Q_{ads} (mg/g)								
					RPS			PST1			PST2		
No	A	B	C	D	E.v.	P.v.	R.	E.v.	P.v.	R.	E.v.	P.v.	R.
1	+1	-1	+1	+1	6.15	7.32	1.17	7.43	8.69	1.26	7.93	10.91	2.98
2	+1	-1	+1	-1	7.42	8.98	1.56	8.95	10.12	1.17	15.35	15.49	0.14
3	0	0	-1	0	0.01	0.84	0.83	1.22	3.00	1.78	0.30	2.18	1.88
4	0	0	0	0	1.20	2.13	0.93	2.60	2.78	0.18	0.60	1.83	1.23
5	-1	+1	+1	+1	0.01	1.44	1.43	0.01	2.29	2.28	0.16	2.68	2.52
6	-1	-1	-1	-1	0.06	1.95	1.89	2.68	4.19	1.51	2.12	4.26	2.14
7	0	+1	0	0	1.43	3.56	2.13	1.56	2.81	1.25	2.06	3.05	0.99
8	-1	0	0	0	0.03	0.31	0.28	0.44	1.08	0.64	0.16	0.60	0.44
9	+1	+1	-1	+1	1.57	2.25	0.68	2.26	3.01	0.75	1.50	2.88	1.38
10	-1	-1	-1	+1	0.40	1.74	1.34	1.18	2.12	0.94	1.08	2.95	1.87
11	+1	+1	+1	-1	3.27	4.31	1.04	1.00	2.69	1.69	0.09	3.47	3.38
12	+1	-1	-1	-1	6.19	7.01	0.82	8.80	9.14	0.34	9.53	12.25	2.72
13	-1	+1	-1	-1	0.38	1.46	1.08	0.19	1.55	1.36	0.04	2.31	2.27
14	0	-1	0	0	5.33	5.31	-0.02	5.39	6.58	1.19	5.14	8.25	3.11
15	0	0	0	+1	1.74	3.04	1.3	2.33	3.13	0.8	0.93	2.39	1.46
16	-1	+1	+1	-1	0.01	1.16	1.15	1.52	1.94	0.42	0.01	1.94	1.93
17	-1	-1	+1	+1	0.41	1.71	1.3	1.39	2.46	1.07	1.10	3.19	2.09
18	+1	+1	+1	+1	4.75	5.66	0.91	2.85	3.86	1.01	0.16	2.46	2.3
19	0	0	0	0	0.92	2.13	1.21	2.07	2.78	0.71	0.57	1.83	1.26
20	0	0	0	-1	3.02	3.82	0.8	1.97	3.61	1.64	0.91	3.55	2.64
21	-1	+1	-1	+1	0.20	1.56	1.36	0.72	2.02	1.3	0.26	4.57	4.31
22	-1	-1	+1	-1	0.17	1.74	1.57	2.77	4.65	1.88	2.16	6.03	3.87
23	0	0	0	0	2.08	2.13	0.05	2.47	2.78	0.31	0.61	1.83	1.22
24	0	0	+1	0	0.48	1.76	1.28	2.95	3.62	0.67	0.64	2.86	2.22
25	+1	-1	-1	+1	3.39	5.17	1.78	5.79	7.83	2.04	6.68	9.19	2.51
26	+1	0	0	0	2.45	4.27	1.82	2.51	4.31	1.8	0.82	4.48	3.66
27	+1	+1	-1	-1	2.17	3.80	1.63	0.40	1.79	1.39	0.01	2.36	2.35

E.v.: Experimental values, P.v.: Predicted values, R: Residuals

Table S6. Analysis of variance of the amount of IC adsorbed by RBS material

Source	Df	SS	MS	F-ratio	P-value
A	1	1.47347	1.47347	38.43	0.0000*
B	1	1.5488	1.5488	40.39	0.0000*
C	1	0.0117556	0.0117556	0.31	0.5900
D	1	0.47045	0.47045	12.27	0.0044*
A^2	1	0.00433038	0.00433038	0.11	0.7426
AB	1	1.32826	1.32826	34.64	0.0001*
AC	1	0.104006	0.104006	2.71	0.1255
AD	1	0.479556	0.479556	12.51	0.0041*
B^2	1	0.237583	0.237583	6.20	0.0285*
BC	1	0.0612562	0.0612562	1.60	0.2303
BD	1	0.322056	0.322056	8.40	0.0134*
C^2	1	0.0029661	0.0029661	0.08	0.7857
CD	1	0.00050625	0.00050625	0.01	0.9104
D^2	1	0.0122294	0.0122294	0.32	0.5827
		R^2 = 93.4225 % Adjusted R^2 = 85.7488 %			

*Significant; Df = degree of freedom; SS = sum of squares MS = mean square; adjR^2=adjusted R^2

Table S7. Analysis of variance of the amount of IC adsorbed BST1 material

Source	Df	SS	MS	F-ratio	P-value
A	1	2.50134	2.50134	68.31	0.0000*
B	1	3.24276	3.24276	88.56	0.0000*
C	1	0.00605	0.00605	0.17	0.6915
D	1	0.22445	0.22445	6.13	0.0292*
A^2	1	0.00000714286	0.00000714286	0.00	0.9891
AB	1	1.7689	1.7689	48.31	0.0000*
AC	1	0.0036	0.0036	0.10	0.7592
AD	1	0.1444	0.1444	3.94	0.0704
B^2	1	0.625829	0.625829	17.09	0.0014*
BC	1	0.000625	0.000625	0.02	0.8982
BD	1	0.172225	0.172225	4.70	0.0509
C^2	1	0.0132071	0.0132071	0.36	0.5593
CD	1	0.002025	0.002025	0.06	0.8180
D^2	1	0.00257857	0.00257857	0.07	0.7952
		R^2 = 95.4269 % Adjusted R^2 = 90.0917 %			

*Significant; Df = degree of freedom; SS = sum of squares MS = mean square; adjR^2=adjusted R^2

Table S8. Analysis of variance of the amount of IC adsorbed by BST2 material

**Source**	Df	SS	MS	F-ratio	P-value
A	1	4.54009	4.54009	58.38	0.0000*
B	1	5.80269	5.80269	74.62	0.0000*
C	1	0.0997556	0.0997556	1.28	0.2795
D	1	0.961422	0.961422	12.36	0.0043*
A^2	1	0.00237178	0.00237178	0.03	0.8643
AB	1	1.5376	1.5376	19.77	0.0008*
AC	1	0.070225	0.070225	0.90	0.3607
AD	1	0.540225	0.540225	6.95	0.0217*
B^2	1	1.12137	1.12137	14.42	0.0025*
BC	1	0.09	0.09	1.16	0.3032
BD	1	0.6241	0.6241	8.03	0.0151*
C^2	1	0.0022575	0.0022575	0.03	0.8675
CD	1	0.050625	0.050625	0.65	0.4355
D^2	1	3.52734E-7	3.52734E-7	0.00	0.9983
		R^2 = 94.7797 % Adjusted R^2 = 88.6893 %			

*Significant; Df = degree of freedom; SS = sum of squares MS = mean square; adjR^2=adjusted R^2

Table S9. Analysis of variance of the amount of IC adsorbed by RPS material

**Source**	Df	SS	MS	F-ratio	P-value
A	1	70.7653	70.7653	103.31	0.0000*
B	1	13.7463	13.7463	20.07	0.0008*
C	1	3.82722	3.82722	5.59	0.0358*
D	1	0.920272	0.920272	1.34	0.2690
A^2	1	0.381884	0.381884	0.56	0.4696
AB	1	7.49391	7.49391	10.94	0.0063*
AC	1	4.74151	4.74151	6.92	0.0219*
AD	1	0,805506	0.805506	1.18	0.2995
B^2	1	7.91672	7.91672	11.56	0.0053*
BC	1	0.00950625	0.00950625	0.01	0.9082
BD	1	1.09726	1.09726	1.60	0.2297
C^2	1	4.89966	4.89966	7.15	0.0202*
CD	1	0.851006	0.851006	1.24	0.2868
D^2	1	1.46434	1.46434	2.14	0.1694
		R^2 = 93.4812 % Adjusted R^2 = 85.876 %			

*Significant; Df = degree of freedom; SS = sum of squares MS = mean square; adjR^2=adjusted R^2

Table S10. Analysis of variance of the amount of IC adsorbed by PST1 material

**Source**	Df	SS	MS	F-ratio	P-value
A	1	47.0127	47.0127	89.14	0.0000*
B	1	63.732	63.732	120.84	0.0000*
C	1	1.76094	1.76094	3.34	0.0926
D	1	1.0368	1.0368	1.97	0.1862
A^2	1	0.998521	0.998521	1.89	0.1940
AB	1	22.2784	22.2784	42.24	0.0000*
AC	1	0.265225	0.265225	0.50	0.4918
AD	1	0.5776	0.5776	1.10	0.3159
B^2	1	4.87471	4.87471	9.24	0.0103*
BC	1	0.0049	0.0049	0.01	0.9248
BD	1	6.42622	6.42622	12.18	0.0045*
C^2	1	0.000444533	0.000444533	0.00	0.9773
CD	1	0.0121	0.0121	0.02	0.8821
D^2	1	0.00691358	0.00691358	0.01	0.9107
		R^2 = 95.9566 %	Adjusted R^2 = 91.2393 %		

*Significant; Df = degree of freedom; SS = sum of squares MS = mean square; adjR^2=adjusted R^2

Table S11. Analysis of variance of the amount of IC adsorbed by PST2 material

**Source**	Df	SS	MS	F-ratio	P-value
A	1	67.9778	67.9778	36.49	0.0001*
B	1	121.68	121.68	65.31	0.0000*
C	1	2.05369	2.05369	1.10	0.3144
D	1	6.03202	6.03202	3.24	0.0971
A^2	1	0.242998	0.242998	0.13	0.7243
AB	1	62.9642	62.9642	33.80	0.0001*
AC	1	2.1609	2.1609	1.16	0.3027
AD	1	3.04502	3.04502	1.63	0.2253
B^2	1	20.1974	20.1974	10.84	0.0064*
BC	1	4.5369	4.5369	2.44	0.1446
BD	1	12.7806	12.7806	6.86	0.0224*
C^2	1	0.275646	0.275646	0.15	0.7072
CD	1	2.3104	2.3104	1.24	0.2872
D^2	1	0.0386459	0.0386459	0.02	0.8879
		R^2 = 93. 5205 %	Adjusted R^2 = 85. 9611 %		

*Significant; Df = degree of freedom; SS = sum of squares MS = mean square; adjR^2=adjusted R^2

Table S12. Data from the Redlich-Peterson, Hill, Kaln and Toth isotherms for the sorption of IC by RBS, BST1 and BST2 materials.

Models	Constants	Value	R^2	χ^2	Errors			
					REQM	HYBRID	ERM	SEA
				IC/RBS				
	Q_m(mg/g)	5.315						
Hill	K_H(L/g)	8.123	0.991	0.023	0.290	0.395	0.069	0.104
	n_H	0.802						
	Q_{max}(mg/g)	3.821					0.154	0.231
Kahn	b_K(L/g)	0.120	0.991	0.023	0.287	0.385		
	a_K	0.885						
Redlich-Peterson	A(L/g)	549.67						
	B(L/mg)	797.91						
	β	0.495	0.993	0.018	0.256	0.306	0.009	0.014
	Q(mg/g)	1.016						
Toth	K_e	1.196	0.992	0.019	0.264	0.326	0.042	0.063
	N	0.772						
				IC/BST1				
	Q_m(mg/g)	222.073						
Hill	K_H(L/g)	490.316						
	n_H	0.694	0.987	0.039	0.387	0.657	0.205	0.328
	Q_{max}(mg/g)	4.296						
Kahn	b_K(L/g)	0.060	0.981	0.054	0.456	0.913	0.706	1.127
	a_K	0.461						
Redlich-Peterson	A(L/g)	39856.8						
	B(L/mg)	84062.51						
	β	0.327	0.987	0.038	0.384	0.648	0.141	0.226
	Q(mg/g)	0.992						
Toth	K_e	3.557	0.984	0.046	0.420	0.773	0.274	0.438
	N	0.698						
				IC/BST2				
	Q_m(mg/g)	4.985						
Hill	K_H(L/g)	2.329	0.986	0.050	0.512	0.845	0.114	0.248
	n_H	0.836						
	Q_{max}(mg/g)	0.664						
Kahn	b_K(L/g)	12.959	0.995	0.016	0.291	0.273	0.179	0.389
	a_K	0.660						
Redlich-Peterson	A(L/g)	66023.6						
	B(L/mg)	47225.6						
	β	0.606	0.996	0.011	0.247	0.196	0.029	0.064
	Q(mg/g)	3.913						
Toth	K_e	3.739	0.991	0.033	0.415	0.557	0.250	0.543
	N	0.944						

Table S13. Data from the Redlich-Peterson, Hill, Kaln and Toth isotherms for the sorption of IC by RPS, PST1 and PST2 materials.

Models	Constants	Value	R^2	χ^2	Errors			
					REQM	HYBRID	ERM	SEA
				IC/RPS				
Hill	Q_m(mg/g)	11.589	0.984	0.144	1.260	2.410	0.250	1.157
	K_H(L/g)	0.935						
	n_H	0.929						
Kahn	Q_{max}(mg/g)	3.369	0.990	0.085	0.969	1.427	0.389	1.795
	b_K(L/g)	19.308						
	a_K	0.787						
Redlich-Peterson	A(L/g)	86331.8	0.996	0.032	0.602	0.549	0.052	0.243
	B(L/mg)	14292.8						
	β	0.768						
Toth	Q(mg/g)	6.039	0.988	0.100	1.048	1.666	0.928	4.283
	K_e	0.094						
	N	0.876						
				IC/PST1				
Hill	Q_m(mg/g)	15.992	0.991	0.054	0.674	0.915	0.131	0.458
	K_H(L/g)	3.388						
	n_H	0.381						
Kahn	Q_{max}(mg/g)	2.901	0.985	0.091	0.870	1.527	0.255	0.886
	b_K(L/g)	6.499						
	a_K	0.799						
Redlich-Peterson	A(L/g)	67664.3	0.992	0.047	0.626	0.790	0.032	0.112
	B(L/mg)	18096.3						
	β	0.754						
Toth	Q(mg/g)	3.739	0.986	0.081	0.820	1.357	0.586	2.037
	K_e	0.100						
	N	0.868						
				IC/PST2				
Hill	Q_m(mg/g)	17.589	0.994	0.049	0.767	0.821	0.080	0.405
	K_H(L/g)	1.354						
	n_H	0.443						
Kahn	Q_{max}(mg/g)	2.274	0.995	0.045	0.740	0.763	0.016	0.083
	b_K(L/g)	194.024						
	a_K	0.779						
Redlich-Peterson	A(L/g)	63249	0.997	0.027	0.572	0.456	0.047	0.240
	B(L/mg)	8838.12						
	β	0.767						
Toth	Q(mg/g)	7.338	0.995	0.045	0.734	0.750	0.046	0.009
	K_e	0.009						
	N	0.884						
